# Sky Blue and Yellow Cluster Light-Emitting Diodes Based on Asymmetric Cu_4_I_4_ Nanocubes

**DOI:** 10.34133/research.0005

**Published:** 2022-12-15

**Authors:** Nan Zhang, Lei Qu, Huan Hu, Ran Huo, Yushan Meng, Chunbo Duan, Jing Zhang, Chunmiao Han, Guohua Xie, Hui Xu

**Affiliations:** ^1^ Key Laboratory of Functional Inorganic Material Chemistry (Ministry of Education) and School of Chemistry and Material Science, Heilongjiang University, 74 Xuefu Road, Harbin 150080, P. R. China.; ^2^ Hubei Key Lab on Organic and Polymeric Optoelectronic Materials, Department of Chemistry, Wuhan University, Wuhan 430072, P. R. China.

## Abstract

Controllably optimizing excited-state characteristics is crucial for luminescent nanoclusters but remains a formidable challenge. Herein, we report an effective “ligand-induced asymmetrization” strategy for constructing thermally activated delayed fluorescence-featured cubic Cu_4_I_4_ nanoclusters with asymmetric configurations, named [tBCzDBFDP]_2_Cu_4_I_4_ and [PTZDBFDP]_2_Cu_4_I_4_. Through changing 3,6-di-*tert*-butyl-carbazole (tBCz) to phenothiazine (PTZ) with a stronger electron-donating effect, emission color is tuned from greenish blue of [tBCzDBFDP]_2_Cu_4_I_4_ to yellow of [PTZDBFDP]_2_Cu_4_I_4_, as well as the triplet locally excited state of the former to the triplet charge transfer state of the latter. Temperature-correlated spectroscopic investigation indicates that in terms of triplet quenching suppression, [tBCzDBFDP]_2_Cu_4_I_4_ is superior to [PTZDBFDP]_2_Cu_4_I_4_, in accord with the stabilities of their triplet locally excited state and triplet charge transfer state. As a consequence, these asymmetric Cu_4_I_4_ nanocubes endowed their cluster light-emitting diodes with the external quantum efficiencies beyond 12% for sky blue and 8% for yellow. These results suggest the significance and effectiveness of ligand engineering for optoelectronic nanoclusters.

## Introduction

Luminescent nanoclusters emerge rapidly in recent decades [1–3]. This kind of material features inorganic nano-skeletons stabilized with organic ligands, thus combining the advantages of organic molecules with high luminescence efficiencies and processibility, and inorganic motifs with high rigidity, unique electronic structures, and excellent thermo- and photostability [1,2,4]. The structural diversity of nanoclusters provides flexibility in exactly modulating shape, size, organic–inorganic ratio, and so on [5–7]. Therefore, luminescent nanoclusters are widely used in bioimaging [8], sensing/detection [9–11], light conversion [12–14], etc. For such organic–inorganic hybrids, functions of ligands are already beyond stabilizing cluster cores, since it is demonstrated that radiative transitions of clusters are predominantly attributed to ligand-centered excited-state components, e.g., intraligand charge transfer(ILCT), counterion-to-ligand charge transfer(XLCT), and ligand locally excited state [15]. Furthermore, our recent study on cluster semiconductors indicated the significant contributions of aromatic ligands to electrical properties [16]. Therefore, ligand engineering provides an effective approach to modulate the comprehensive properties of nanoclusters.

However, the applications of cluster materials in optoelectronic devices still face a big challenge in multifunctional integrity. For example, until now, the electroluminescence (EL) performance of cluster light-emitting diodes (CLEDs) is still far behind that of other kinds of light-emitting devices. In particular, the external quantum efficiencies (EQE, η_EQE_) of CLEDs were mostly less than 10% [17], in contrast to the state-of-the-art value of ~20% for organic light-emitting diodes [18,19], quantum-dot light-emitting diodes [20], and perovskite light-emitting diodes [21,22]. On one hand, the existence of triplet quenching states, especially the triplet cluster-centered state [23–26], makes photoluminescence quantum yield (ϕ_PL_) commonly less than 50% [7,27–30]. On the other hand, despite improving stabilities, rigid cluster structures render aggregation-induced quenching and poor film formability [31]. Therefore, most CLEDs adopted low doping concentrations within 10%, which not only reduces optimization space but also increases fabrication difficulty [32,33].

Copper clusters hold a promise for large-scale luminescent applications, owing to their high ϕ_PL_, low cost, and environmental friendliness [34]. More importantly, phosphine-chelated copper nanoclusters reveal dominant EL performance among cluster emitters [17,35]. Our group demonstrated the first Cu_4_I_4_-based white CLED, despite a low η_EQE_ of 0.7% [36], which already exceeded the values of gold, silver [33], and molybdenum [32] cluster-based devices. Through introducing donor groups in bidentate phosphine ligand, we recently maximized ligand-centered excited-state components to achieve the highest η_EQE_ of 7.9% among Cu_4_I_4_ clusters [37]. Olaru et al. [38] reported another cationic green Cu_4_ cluster with a record η_EQE_ of 11.0% for copper CLEDs. Nonetheless, copper CLEDs still face the same challenges in multifunction balance and quenching suppression, as well as full-color emission.

Herein, an effective “ligand-induced asymmetrization” strategy is demonstrated with 2 diphosphine chelated Cu_4_I_4_ clusters, namely, [tBCzDBFDP]_2_Cu_4_I_4_ and [PTZDBFDP]_2_Cu_4_I_4_, whose ligands are respectively monofunctionalized with 3,6-di-*tert*-butyl-carbazole (tBCz) and phenothiazine (PTZ) (Schemes S1 and S2). Both clusters reveal the ligand-centered excited states with negligible single-triplet splitting energies (Δ*E*_ST_), rendering their typical thermally activated delayed fluorescence (TADF) features. Using PTZ with a stronger donating effect instead of tBCz makes the emission peak wavelength of [PTZDBFDP]_2_Cu_4_I_4_ shift to ~530 nm, corresponding to yellow color, compared to the greenish blue emission of [tBCzDBFDP]_2_Cu_4_I_4_ that peaked at ~495 nm. Enhanced ILCT further induces stronger concentration dependence of the emission properties for [PTZDBFDP]_2_Cu_4_I_4_. Nonetheless, dispersing the clusters in a host matrix (bis-4-(*N*-carbazolyl)phenyl)phenylphosphine oxide [BCPO]), [tBCzDBFDP]_2_Cu_4_I_4_ and [PTZDBFDP]_2_Cu_4_I_4_ achieved state-of-the-art ϕ_PL_ values of 68% and 45%, and state-of-the-art η_EQE_ values of 12.2% and 8.6% for copper CLEDs, respectively, at extremely high doping concentrations of 40% and 30%.

## Results

### Molecular design, structures, and theoretical simulation

**Fig. 1.**
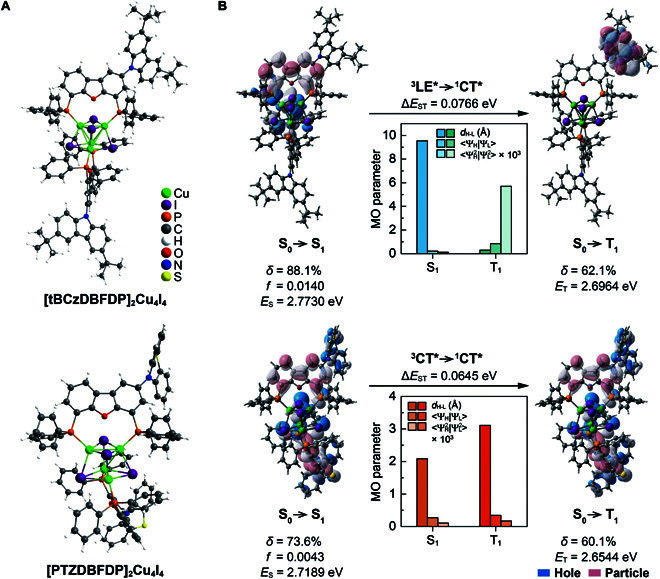
Molecular structure and electronic characteristics of [tBCzDBFDP]_2_Cu_4_I_4_ and [PTZDBFDP]_2_Cu_4_I_4_. (A) Asymmetric molecular structures of [tBCzDBFDP]_2_Cu_4_I_4_ and [PTZDBFDP]_2_Cu_4_I_4_. (B) Time-dependent density functional theory analysis of the singlet and triplet excitations of the clusters. “Hole” and “particle” are the highest occupied and lowest unoccupied natural transition orbitals, respectively. S_0_, S_1_, and T_1_ refer to the ground and the first singlet and triplet excited states, respectively. LE and CT refer to locally excited and charge transfer states, respectively. *E*, δ, *f*, and Δ*E*_ST_ refer to energy level, contribution weight, oscillator strength, and singlet-triplet splitting energy, respectively. *d*_H-L_, 〈*Ψ*_H_|*Ψ*_L_〉, and $\langle \Psi_{H}^{2}|\Psi_{L}^{2}\rangle$ are distance, wave function integral, and electron-cloud-density integral of the “hole” and “particle”, respectively. Superscripts “1” and “3” and subscripts “S” and “T” refer to singlet and triplet states, respectively. Superscript “*” refers to excited state.

Dibenzofuran (DBF) is chosen as the skeleton and electron-withdrawing group, which is disubstituted with diphenylphosphine (DP) at 4,6-positions to form bidentate coordination mode, which is further used to chelate Cu_4_I_4_ nanocubes (Fig. 1A). This orthogonal structure renders extremely high rigidity and stability for the clusters. As a consequence, [tBCzDBFDP]_2_Cu_4_I_4_ and [PTZDBFDP]_2_Cu_4_I_4_ reveal the temperatures of decomposition (*T*_d_) beyond 420 ^o^C (Fig. S1 and Table S1). Furthermore, single tBCz or PTZ is introduced at the 2-position of DBF. Despite plane symmetries of tBCzDBFDP and PTZDBFDP, monofunctionalization of 2 orthogonal ligands leads to asymmetric configurations of the clusters, which effectively suppress intermolecular regular packing. Therefore, no melting points (*T*_m_) are observed, but morphological stabilities of solid-state clusters are verified by their extremely high temperatures of glass transition (*T*_g_) over 290 ^o^C. The clusters have good solubility in common solvents and therefore can be processed with wet approaches, e.g., spin coating, ink printing, and so on, making them competent for large-scale production. The spin-coated films show uniform and smooth morphology with roughness <1 nm (Fig. S2).

Density functional theory simulation shows that for [tBCzDBFDP]_2_Cu_4_I_4_, its first 3 highest occupied molecular orbitals (HOMOs) are localized on Cu_4_I_4_, whose 80% are contributed by iodine atoms (Fig. S3). [PTZDBFDP]_2_Cu_4_I_4_ reveals the similar locations of its HOMO and HOMO-2, except for minor but sustainable dispersions on PTZ groups. Its HOMO-1 is even completely localized on PTZ. Meanwhile, the first 3 lowest unoccupied molecular orbitals (LUMOs) of [PTZDBFDP]_2_Cu_4_I_4_ symmetrically distribute on DBF groups of both ligands. On the contrary, the LUMO and LUMO+1 of [tBCzDBFDP]_2_Cu_4_I_4_ are located on single DBF groups, while its LUMO+2 is instead localized on tBCz. In accord with calculated data, the HOMO and LUMO energy levels of [tBCzDBFDP]_2_Cu_4_I_4_ and [PTZDBFDP]_2_Cu_4_I_4_ are respectively estimated as −5.59/−3.15 eV and −5.42/−3.34 eV with cyclic voltammetry (Fig. S4 and Table S1).

Nature transition orbital (NTO) analyses on the S_0_→S_1_ and S_0_→T_1_ excitations show that the “hole” and “particle” of the singlet excitations for [tBCzDBFDP]_2_Cu_4_I_4_ are respectively localized on iodine atoms and the DBF group, corresponding to the XLCT-predominant first singlet excited state (S_1_) (Fig. 1B). In comparison, despite the same “particle” location on DBF, the “hole” of the singlet excitation for [PTZDBFDP]_2_Cu_4_I_4_ is dispersed on iodine atoms and PTZ groups with ratios of 70% and 30%, respectively, corresponding to the XLCT/ILCT hybrid S_1_ state. Therefore, although the more uniform “hole” distribution of [PTZDBFDP]_2_Cu_4_I_4_ renders its smaller distance between “hole” and “particle” (*d*_H-L_) and larger wave function integral (〈*Ψ*_H_|*Ψ*_L_〉) and electron-cloud-density integral ($\langle \Psi_{H}^{2}|\Psi_{L}^{2}\rangle$) of “hole” and “particle”, its singlet oscillator strength (*f*_S_) is still smaller than that of [tBCzDBFDP]_2_Cu_4_I_4_. The first triplet excited state (T_1_) of [PTZDBFDP]_2_Cu_4_I_4_ also features XLCT/ILCT hybrid, nearly identical to its S_1_ state. On the contrary, the T_1_ state of [tBCzDBFDP]_2_Cu_4_I_4_ is a locally excited state with overlapped “hole” and “particle” on tBCz. Therefore, intramolecular charge transfer (CT) interaction in [PTZDBFDP]_2_Cu_4_I_4_ is stronger, leading to the reduced S_1_ and T_1_ energy levels and Δ*E*_ST_.

### Photophysical properties

**Fig. 2.**
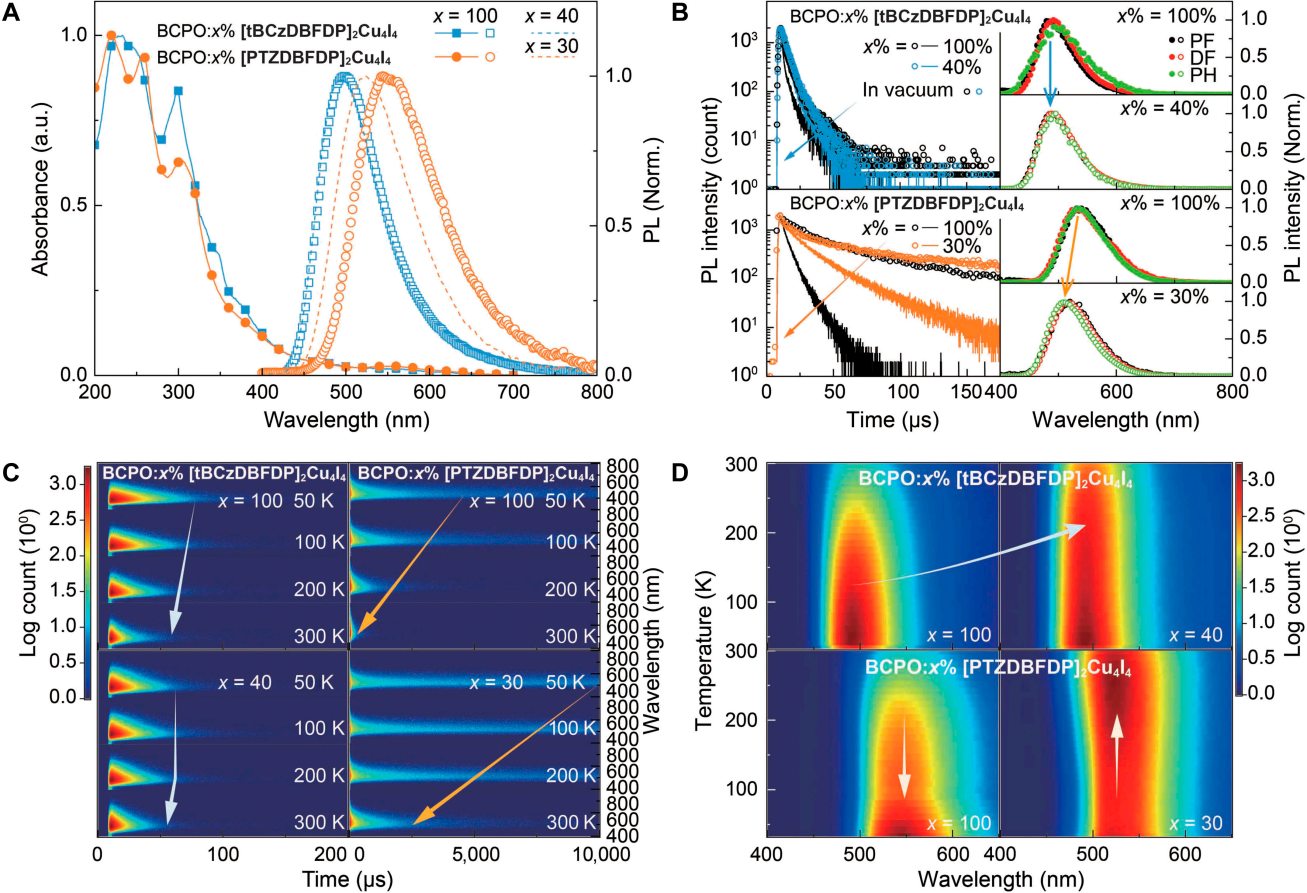
Photophysical properties of [tBCzDBFDP]_2_Cu_4_I_4_ and [PTZDBFDP]_2_Cu_4_I_4_. (A) Electronic absorption and steady-state photoluminescence (PL) spectra of BCPO:*x*% [tBCzDBFDP]_2_Cu_4_I_4_ and [PTZDBFDP]_2_Cu_4_I_4_ films. BCPO is bis-4-(*N*-carbazolyl)phenyl)phenylphosphine oxide used as matrix. *x* = 100 for neat films (hollow symbols), 40 for the [tBCzDBFDP]_2_Cu_4_I_4_-doped film (blue dashed line), and 30 for the [tBCzDBFDP]_2_Cu_4_I_4_-doped film (orange dashed line). a.u., arbitrary units. (B) Time decays (left) of BCPO:*x*% [tBCzDBFDP]_2_Cu_4_I_4_ and [PTZDBFDP]_2_Cu_4_I_4_ films in atmosphere (lines) and vacuum (hollow symbols), and prompt fluorescence (PF), delayed fluorescence (DF), and phosphorescence (PH) spectra. (C) TRES of BCPO:*x*% [tBCzDBFDP]_2_Cu_4_I_4_ and [PTZDBFDP]_2_Cu_4_I_4_ films recorded at 50, 100, 200, and 300 K, respectively. (D) Contours of temperature-dependent steady-state PL spectra for BCPO:*x*% [tBCzDBFDP]_2_Cu_4_I_4_ and [PTZDBFDP]_2_Cu_4_I_4_ films recorded at the temperature range of 20 to 300 K with an interval of 10 K.

In dilute solutions, the clusters display similar electronic absorption bands that peaked around 230, 290, and 350 nm, corresponding to π→π*, n→π*, and ILCT transitions, respectively (Fig. S5). The absorption bands of ligands and clusters are similar, verifying the antenna effect of ligands in energy absorption of clusters (Fig. S6a). Nonetheless, in contrast to the CT-predominant excitation of PTZDBFDP, excitation bands of tBCzDBFDP are composed of locally excited and CT components, due to the weaker electron-donating effect of carbazole. The ligand-originated excitation bands are secondary in the spectra of clusters, in which a long-wavelength band attributed to MLCT, XLCT, and coordination-enhanced ILCT becomes primary. Compared to tBCzDBFDP, the stronger intramolecular CT in PTZDBFDP is verified by its emission red-shifted by 80 nm (Fig. S6b). Coordination with Cu_4_I_4_ also enhances CT interactions, resulting in the similar red shifts by 64 and 37 nm for [tBCzDBFDP]_2_Cu_4_I_4_ and [PTZDBFDP]_2_Cu_4_I_4_, respectively. In neat films, all the absorption peaks are enhanced due to intermolecular interactions (Fig. 2a). In particular, weak long absorption tails are observed in the range of 450 to 600 nm, ascribed to XLCT transitions. The steady-state photoluminescence (PL) spectrum of the neat [tBCzDBFDP]_2_Cu_4_I_4_ film nearly overlapped with that of BCPO:40% [tBCzDBFDP]_2_Cu_4_I_4_, whose peak wavelengths are at ~495 nm (Table). In contrast, the neat [PTZDBFDP]_2_Cu_4_I_4_ film reveals orange emission that peaked at 547 nm, but the peak wavelength of BCPO:30% [PTZDBFDP]_2_Cu_4_I_4_ shifts to 523 nm, corresponding to yellow emission. After doping in BCPO matrix, [tBCzDBFDP]_2_Cu_4_I_4_ and [PTZDBFDP]_2_Cu_4_I_4_ realize high ϕ_PL_ values of 68% and 45%, respectively, which are outstanding among Cu_4_I_4_ clusters.

**Table.** Physical properties of the Cu_4_I_4_ clusters

Cluster	λ_PL_ (nm)	S_1_ (eV)	T_1_ (eV)	Δ*E*_ST_^a^ (eV)	ϕ_PL_^b^ (%)	η_EQE_^c^ (%)	η_EUE_^d^ (%)
[tBCzDBFDP]_2_Cu_4_I_4_	495^e^	2.77^f^	2.70^f^	0.07^f^	68^eg^	12.2^g^	81
498^eg^	2.55^h^	2.50^i^	0.05^hi^				
[PTZDBFDP]_2_Cu_4_I_4_	547^e^	2.72^f^	2.65^f^	0.07^f^	45^eg^	8.6^g^	76
523^eg^	2.38^h^	2.34^i^	0.04^hi^				

^a^Singlet-triplet splitting. ^b^Photoluminescence quantum yields of BCPO:40% [tBCzDBFDP]_2_Cu_4_I_4_ and BCPO:30% [PTZDBFDP]_2_Cu_4_I_4_ films. ^c^External quantum efficiencies of CLEDs based on BCPO:40% [tBCzDBFDP]_2_Cu_4_I_4_ and BCPO:30% [PTZDBFDP]_2_Cu_4_I_4_. ^d^Exciton utilization efficiencies of CLEDs based on BCPO:40% [tBCzDBFDP]_2_Cu_4_I_4_ and BCPO:30% [PTZDBFDP]_2_Cu_4_I_4_. ^e^In film. ^f^Gaussian simulation results of single molecules. ^g^For BCPO:40% [tBCzDBFDP]_2_Cu_4_I_4_ and BCPO:30% [PTZDBFDP]_2_Cu_4_I_4_. ^h^Estimated according to 0–0 transition of prompt fluorescence. ^i^Estimated with 0–0 transition of phosphorescence.

In dilute toluene, [tBCzDBFDP]_2_Cu_4_I_4_ and [PTZDBFDP]_2_Cu_4_I_4_ reveal the microsecond time decays, in comparison to nanosecond decays of the corresponding ligands (Fig. S7). [tBCzDBFDP]_2_Cu_4_I_4_ and [PTZDBFDP]_2_Cu_4_I_4_ neat films reveal similar time decay curves in the microsecond range (Fig. 2B). It is noted that in vacuum, lifetimes of the films are markedly elongated, indicating that the emissions originated from triplet states. Moreover, emission of the [PTZDBFDP]_2_Cu_4_I_4_ film is significantly more sensitive to oxygen than the [tBCzDBFDP]_2_Cu_4_I_4_ film. It is shown that lifetimes of the BCPO:*x*% cluster are basically in reverse proportion to *x*, since BCPO matrix can also alleviate collision-induced concentration quenching, e.g., triplet–triplet annihilation (Figs. S8 and S9). BCPO matrix effectively restrains oxygen exposure-induced quenching. Therefore, time decays of the BCPO:40% [tBCzDBFDP]_2_Cu_4_I_4_ film in air and vacuum nearly overlapped. However, BCPO:30% [PTZDBFDP]_2_Cu_4_I_4_ still reveals considerably elongated decay in vacuum. It means that the triplet locally excited state of [tBCzDBFDP]_2_Cu_4_I_4_ is more stable than the triplet CT state of [PTZDBFDP]_2_Cu_4_I_4_. With the time-resolved method, prompt fluorescence, delayed fluorescence, and phosphorescence spectra of the clusters almost overlapped, corresponding to near-zero Δ*E*_ST_ within 0.05 eV (Table S1), which are in accord with NTO results. Compared to tBCzDBFDP with vibrational shoulder peak, the prompt fluorescence profile of [tBCzDBFDP]_2_Cu_4_I_4_ is markedly narrowed, owing to enhanced molecular rigidity.

Time-resolved emission spectra (TRES) of [tBCzDBFDP]_2_Cu_4_I_4_ in dilute toluene solutions further demonstrate the rigid enhancement by cluster formation markedly narrow profile (Fig. S11). Temperature-dependent TRES (TDTRES) of BCPO:*x*% [tBCzDBFDP]_2_Cu_4_I_4_ films show that, at *x* = 100, increasing temperature from 50 to 300 K makes emission contours gradually shortened from 100 to 50 μs, while at *x* = 40, TDTRES is nearly unchanged from 50 to 200 K, but shortened at 300 K, indicating the balance between triplet quenching and radiation acceleration and the effect of BCPO on quenching suppression (Fig. 2C and Figs. S8 and S9). In contrast, for BCPO:*x*% [PTZDBFDP]_2_Cu_4_I_4_ films, besides alleviated triplet quenching at *x* = 30, increasing temperature induces markedly shortened contours from 10 ms to hundreds of microseconds, revealing radiation shift from the forbidden triplet state to allow singlet state at 300 K for [PTZDBFDP]_2_Cu_4_I_4_. Temperature-correlated PL spectra further show that compared to neat films, PL intensities of BCPO:40% [tBCzDBFDP]_2_Cu_4_I_4_ at 200 to 300 K largely increase, manifesting the effect of BCPO host on quenching suppression and radiation facilitation (Fig. 2D and Fig. S10). The influence of BCPO on [PTZDBFDP]_2_Cu_4_I_4_-based films is more remarkable. Compared to largely decreased PL intensities of neat films at temperature >50 K, BCPO:30% [PTZDBFDP]_2_Cu_4_I_4_ achieves the bigger PL intensities in the range of 200 to 300 K, further manifesting its TADF-predominant radiative feature. As a consequence, ϕ_PL_ values of [tBCzDBFDP]_2_Cu_4_I_4_ and [PTZDBFDP]_2_Cu_4_I_4_ are more than twice of those of their ligands.

### CLED performance

**Fig. 3.**
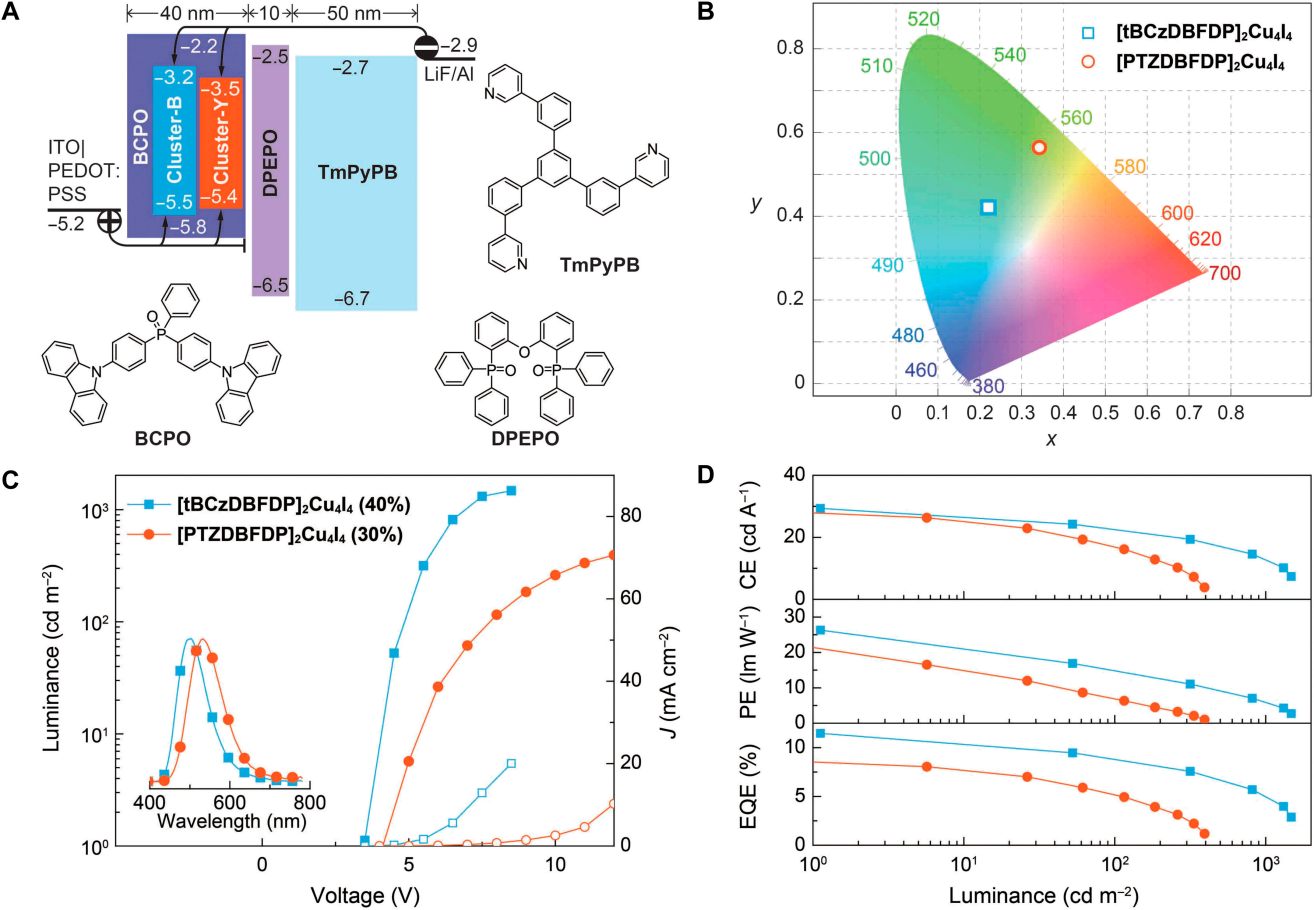
EL performance of CLEDs. (A) Device structure and energetic diagram of spin-coated CLEDs and molecular structures of employed BCPO host, bis(2-(diphenylphosphino)phenyl) ether oxide (DPEPO) as exciton-blocking layer, and 1,3,5-tri[(3-pyridyl)-phen-3-yl]benzene (TmPyPB) as electron-transporting layer. Carrier injection, transportation, and recombination routines were marked with arrows. ITO, indium tin oxide. PEDOT:PSS, poly(3,4-ethylenedioxythiophene):poly(styrenesulfonate). (B) Luminance–current density (*J*)–voltage curves and EL spectra (inset) of the devices with optimal doping concentrations as 40% for [tBCzDBFDP]_2_Cu_4_I_4_ and 30% for [PTZDBFDP]_2_Cu_4_I_4_, respectively. (C) Efficiency–luminance curves of [tBCzDBFDP]_2_Cu_4_I_4_-based (square) and [PTZDBFDP]_2_Cu_4_I_4_-based (circle) CLEDs.

CLEDs based on [tBCzDBFDP]_2_Cu_4_I_4_ and [PTZDBFDP]_2_Cu_4_I_4_ were fabricated through spin coating with a conventional trilayer structure of indium tin oxide|poly(3,4-ethylenedioxythiophene): poly(styrenesulfonate) (40 nm)|BCPO:*x*% cluster (40 nm)|bis(2-(diphenylphosphino)phenyl) ether oxides (10 nm)|1,3,5-tri[(3-pyridyl)-phen-3-yl]benzene (50 nm)|LiF (1 nm)|Al (100 nm) (Fig. 3A). The doping concentration *x*% was tuned to achieve the optimal device performance (Figs. S11 and S12). It is shown that increasing *x*% induced EL emission red-shifted by 4 to 12 nm (Table S2). At the optimal *x* of 40 and 30 for [tBCzDBFDP]_2_Cu_4_I_4_ and [PTZDBFDP]_2_Cu_4_I_4_, respectively, their devices achieved the highest performance. The [tBCzDBFDP]_2_Cu_4_I_4_-based device displayed greenish blue emission that peaked at 490 to 500 nm with Commission Internationale de lEclairage coordinates of (0.21 ± 0.1, 0.43 ± 0.1), while the yellow EL emission from [PTZDBFDP]_2_Cu_4_I_4_ peaked at 532 nm, corresponding to Commission Internationale de lEclairage coordinates of (0.34, 0.56) (Fig. 3b). Despite its deeper LUMO and shallower HOMO, the driving voltages of [PTZDBFDP]_2_Cu_4_I_4_-based devices were still higher than those of [tBCzDBFDP]_2_Cu_4_I_4_-based analogs, due to relative low radiative efficiency (Fig. 3c). It indicates that host–dopant energy transfer and exciton migration are the predominant EL mechanisms. The CLEDs revealed the duration similar to previously reported spin-coated devices of copper-based materials [39], due to the intrinsic disadvantages of the spin-coating technique such as simple stacks and encapsulation.

More importantly, [tBCzDBFDP]_2_Cu_4_I_4_ endowed its CLEDs with state-of-the-art efficiencies up to 29.3 cd A^−1^ for current efficiency (CE, η_CE_), 26.3 lm W^−1^ for power efficiency (CE, η_PE_), and 12.2% for η_EQE_, which are the state-of-the-art values of cyan CLEDs reported so far (Table S3). The maximum efficiencies of [PTZDBFDP]_2_Cu_4_I_4_-based CLEDs also reached 28.1 cd A^−1^, 22.0 lm W^−1^, and 8.6%, respectively, which are markedly higher than those of yellow mononuclear copper complexes [3]. It is noted that compared to the maximum η_EQE_ of ~1% for the parent cluster [DBFDP]_2_Cu_4_I_4_ [36], functionalization with tBCz and PTZ and asymmetric configurations result in efficiency improvements of [tBCzDBFDP]_2_Cu_4_I_4_ and [PTZDBFDP]_2_Cu_4_I_4_ by 12- and 8-fold. Furthermore, compared to its bifuncitonalized congener [DtBCzDBF]_2_Cu_4_I_4_ [37], the ϕ_PL_ of [tBCzDBFDP]_2_Cu_4_I_4_ is similar, but its η_EQE_ was improved by 50%. It indicates that asymmetrical configuration can further reduce the involvement of quenching states in the EL process. In contrast to our recently reported acridine-modified green Cu_4_I_4_ clusters [40], the cyan and yellow PL and EL emissions from [tBCzDBFDP]_2_Cu_4_I_4_ and [PTZDBFDP]_2_Cu_4_I_4_ manifest the significant influence of the electron-donating effect on excited energy levels, which provides a facile approach for constructing full-color CLEDs. Taking the light out-coupling efficiency of indium tin oxide glass as 25%, the exciton utilization efficiency (EUE, η_EUE_) of [tBCzDBFDP]_2_Cu_4_I_4_- and [PTZDBFDP]_2_Cu_4_I_4_-based CLEDs was 81% and 76%, respectively, which were higher than 70% for [DBFDP]_2_Cu_4_I_4_-based analogs [36]. Nonetheless, compared to η_EUE_, the differences between η_EQE_ values of the CLEDs are markedly larger, which is attributed to the big ϕ_PL_ difference of the clusters. Therefore, it is convincing that excited-state optimization for simultaneous radiation acceleration and quenching suppression is the key factor determining both PL and EL performances of copper nanoclusters.

## Discussion

In summary, 2 monofunctionalized diphosphine ligands are developed to demonstrate “ligand-induced asymmetrization” strategy for high-efficiency Cu_4_I_4_ clusters. Incorporating a single tBCz or PTZ group makes ligand-involved transitions predominant in excited states of [tBCzDBFDP]_2_Cu_4_I_4_ and [PTZDBFDP]_2_Cu_4_I_4_, rendering near-zero Δ*E*_ST_ and typical TADF characteristics. Different intensities of electron-donating effects for tBCz and PTZ groups result in not only greenish blue and yellow emissions but also different triplet locally excited state and triplet CT state of the clusters. Nonetheless, both [tBCzDBFDP]_2_Cu_4_I_4_ and [PTZDBFDP]_2_Cu_4_I_4_ achieve high ϕ_PL_ values of 68% and 45%, respectively. In comparison to the parent cluster [DBFDP]_2_Cu_4_I_4_, η_EQE_ values of [tBCzDBFDP]_2_Cu_4_I_4_- and [PTZDBFDP]_2_Cu_4_I_4_-based CLEDs were improved by 12- and 8-fold to new record values of 12.2% and 8.8%, respectively. These results suggest that excited-state modulation by ligand engineering is feasible and crucial for optimizing single-molecule and condensed-state properties of clusters. This work further solidly demonstrates great potentials of copper CLEDs in practical displaying and lighting applications.

## Materials and Methods

Experimental details, thermal properties, density functional theory simulation, electrochemical and photophysical properties, and device performance are included in the Supplementary Materials.

## Data Availability

All other data are available from the authors upon reasonable request.

## Supplementary Materials

Supplementary Materials Scheme S1. Synthetic procedures of [tBCzDBFDP]_2_Cu_4_I_4_. Scheme S2. Synthetic procedures of [PTZDBFDP]_2_Cu_4_I_4_. Fig. S1. TGA and DSC curves of [tBCzDBFDP]_2_Cu_4_I_4_ and [PTZDBFDP]_2_Cu_4_I_4_. Fig. S2. FMO energy levels and contours of [tBCzDBFDP]_2_Cu_4_I_4_ and [PTZDBFDP]_2_Cu_4_I_4_ simulated with the B3LYP/6-31G* method. Fig. S3. CV curves of [tBCzDBFDP]_2_Cu_4_I_4_ and [PTZDBFDP]_2_Cu_4_I_4_ measured at room temperature with the scanning rate of 100 mV s^−1^. Fig. S4. Electronic absorption spectra of [tBCzDBFDP]_2_Cu_4_I_4_ and [PTZDBFDP]_2_Cu_4_I_4_ in dilute dichloromethane (10^−6^ mol l^−1^). Fig. S5. Doping concentration dependence of time decay curves for BCPO:*x*% [tBCzDBFDP]_2_Cu_4_I_4_ films (*x* = 20 to 50 and 100). Fig. S6. Doping concentration dependence of time decay curves for BCPO:*x*% [PTZDBFDP]_2_Cu_4_I_4_ films (*x* = 20 to 50 and 100). Fig. S7. Time decay curves of BCPO:*x*% [tBCzDBFDP]_2_Cu_4_I_4_ (*x* = 40 and 100 for neat film) in the temperature range from 20 to 300 K with an interval of 10 K. Fig. S8. Time decay curves of BCPO:*x*% [PTZDBFDP]_2_Cu_4_I_4_ (*x* = 30 and 100 for neat film) in the temperature range from 20 to 300 K with an interval of 10 K. Fig. S9. PL spectra of BCPO:*x*% [tBCzDBFDP]_2_Cu_4_I_4_ and BCPO:*x*% [PTZDBFDP]_2_Cu_4_I_4_ (*x* = 40 for the former, 30 for the latter, and 100 for neat films) in the temperature range from 20 to 300 K with an interval of 10 K. Fig. S10. (a) EL spectra (inset) and current density (*J*)–voltage–luminance characteristics of BCPO:*x*% [tBCzDBFDP]_2_Cu_4_I_4_-based CLEDs at different *x*. (b) Efficiencies vs. luminance relationships. Fig. S11. (a) EL spectra (inset) and current density (*J*)–voltage–luminance characteristics of BCPO:*x*% [PTZDBFDP]_2_Cu_4_I_4_-based CLEDs at different *x*. (b) Efficiencies vs. luminance relationships. Table S1. Physical properties of the clusters. Table S2. EL performance of CLEDs based on the clusters.
