# Supplementary material for: Sky Blue and Yellow Cluster Light-Emitting Diodes Based on Asymmetric Cu4I4 Nanocubes
Source: Research (Wash D C). 2022 Dec 15;2022:0005. doi: 10.34133/research.0005 (PMC11404315; doi:10.34133/research.0005)
Supplement: Supplementary Materials — Scheme S1. Synthetic procedures of [tBCzDBFDP]2Cu4I4. Scheme S2. Synthetic procedures of [PTZDBFDP]2Cu4I4. Fig. S1. TGA and DSC curves of [tBCzDBFDP]2Cu4I4 and [PTZDBFDP]2Cu4I4. Fig. S2. FMO energy levels and contours of [tBCzDBFDP]2Cu4I4 and [PTZDBFDP]2Cu4I4 simulated with the B3LYP/6-31G* method. Fig. S3. CV curves of [tBCzDBFDP]2Cu4I4 and [PTZDBFDP]2Cu4I4 measured at room temperature with the scanning rate of 100 mV s−1. Fig. S4. Electronic absorption spectra of [tBCzDBFDP]2Cu4I4 and [PTZDBFDP]2Cu4I4 in dilute dichloromethane (10−6 mol l−1). Fig. S5. Doping concentration dependence of time decay curves for BCPO:x% [tBCzDBFDP]2Cu4I4 films (x = 20 to 50 and 100). Fig. S6. Doping concentration dependence of time decay curves for BCPO:x% [PTZDBFDP]2Cu4I4 films (x = 20 to 50 and 100). Fig. S7. Time decay curves of BCPO:x% [tBCzDBFDP]2Cu4I4 (x = 40 and 100 for neat film) in the temperature range from 20 to 300 K with an interval of 10 K. Fig. S8. Time decay curves of BCPO:x% [PTZDBFDP]2Cu4I4 (x = 30 and 100 for neat film) in the temperature range from 20 to 300 K with an interval of 10 K. Fig. S9. PL spectra of BCPO:x% [tBCzDBFDP]2Cu4I4 and BCPO:x% [PTZDBFDP]2Cu4I4 (x = 40 for the former, 30 for the latter, and 100 for neat films) in the temperature range from 20 to 300 K with an interval of 10 K. Fig. S10. (a) EL spectra (inset) and current density (J)–voltage–luminance characteristics of BCPO:x% [tBCzDBFDP]2Cu4I4-based CLEDs at different x. (b) Efficiencies vs. luminance relationships. Fig. S11. (a) EL spectra (inset) and current density (J)–voltage–luminance characteristics of BCPO:x% [PTZDBFDP]2Cu4I4-based CLEDs at different x. (b) Efficiencies vs. luminance relationships. Table S1. Physical properties of the clusters. Table S2. EL performance of CLEDs based on the clusters. [file 0005.f1.docx]

**Supplementary Information**

**Sky-Blue and Yellow Cluster Light-Emitting Diodes Based on Asymmetric Cu_4_I_4_ Nanocubes**

Nan Zhang,^1^ Lei Qu,^1^ Huan Hu,^1^ Ran Huo,^1^ Yushan Meng,^1^ Chunbo Duan,^1^ Jing Zhang,*^1^ Chunmiao Han,^1^ Guohua Xie,*^2^ and Hui Xu*^1^

^1^ Key Laboratory of Functional Inorganic Material Chemistry, Ministry of Education & School of Chemistry and Material Science, Heilongjiang University, 74 Xuefu Road, Harbin 150080 (P. R. China)

^2^ Hubei Collaborative Innovation Centre for Advanced Organic Chemical Materials, Department of Chemistry, Wuhan University, Wuhan 430072, P. R. China.

**Content**

[Experimental Details 2](#_Toc92439427)

[Thermal Properties 12](#_Toc92439428)

[DFT Simulation 13](#_Toc92439429)

[Electrochemical Properties 14](#_Toc92439430)

[Photophysical Properties 15](#_Toc92439431)

[Table S1. Physical properties of the clusters. 20](#_Toc92439432)

[EL Performance 21](#_Toc92439433)

[Table S2. EL performance of CLEDs based on the clusters. 23](#_Toc92439434)

[References 24](#_Toc92439435)

### Experimental Details

*1. Materials and Instruments*

All the reagents and solvents used for the synthesis of the compounds were purchased from Aldrich and Acros companies and used without further purification. ^1^H NMR spectra were recorded using a Varian Mercury plus 400NB spectrometer relative to tetramethylsilane (TMS) as internal standard. Molecular masses were determined by a FINNIGAN LCQ Electro-Spraying Ionization-Mass Spectrometry (ESI-MS), or a MALDI-TOF-MS. Elemental analyses were performed on a Vario EL III elemental analyzer. The crystal suitable for single-crystal XRD analysis was obtained through slowly diffusing hexane into dichloromethane solution of **[DBFDP]_2_Cu_4_I_4_** at room temperature. All diffraction data were collected at 295 K on a Rigaku Xcalibur E diffractometer with graphite monochromatized Mo Kα (λ = 0.71073 Å) radiation in ω scan mode. All structures were solved by direct method and difference Fourier syntheses. Non-hydrogen atoms were refined by full-matrix least-squares techniques on F2 with anisotropic thermal parameters. The hydrogen atoms attached to carbons were placed in calculated positions with C−H = 0.93 Å and U(H) = 1.2Ueq(C) in the riding model approximation. All calculations were carried out with the SHELXL97 program. Absorption and photoluminescence (PL) emission spectra of the target compound were measured using a SHIMADZU UV-3150 spectrophotometer and a SHIMADZU RF-5301PC spectrophotometer, respectively. Thermogravimetric analysis (TGA) and differential scanning calorimetry (DSC) were performed on Shimadzu DSC-60A and DTG-60A thermal analyzers under nitrogen atmosphere at a heating rate of 10 °C min^-1^. Cyclic voltammetric (CV) studies were conducted using an Eco Chemie B. V. AUTOLAB potentiostat in a typical three-electrode cell with a glassy carbon working electrode, a platinum wire counter electrode, and a silver/silver chloride (Ag/AgCl) reference electrode. All electrochemical experiments were carried out under a nitrogen atmosphere at room temperature in dichloromethane. Phosphorescence spectra were measured in dichloromethane using an Edinburgh FLS 1000 fluorescence spectrophotometer at 77 K cooling by liquid nitrogen with a delay of 300 μs using Time-Correlated Single Photon Counting (TCSPC) method with a microsecond pulsed Xenon light source for 10 μs-10 s lifetime measurement, the synchronization photomultiplier for signal collection and the Multi-Channel Scaling Mode of the PCS900 fast counter PC plug-in card for data processing. Cluster based films (20-40 nm) were prepared through spin coating for optical analysis. Photoluminescence quantum yields (PLQY) of these films were measured through a labsphere 1-M-2 (*φ* = 6'') integrating sphere coated by Benflect with efficient light reflection in a wide range of 200-1600 nm, which was integrated with FPLS 920. The absolute PLQY determination of the sample was performed by two spectral (emission) scans, with the emission monochromator scanning over the Rayleigh scattered light from the sample and from a blank substrate. The first spectrum recorded the scattered light and the emission of the sample, and the second spectrum contained the scattered light of Benflect coating. The integration and subtraction of the scattered light parts in these two spectra arrived at the photon number absorbed by the samples (*N*_a_); while, integration of the emission of the samples to calculate the emissive photon number (*N*_e_). Then, the absolute PLQY (*η*) can be estimated according to the equation of *η* = *N*_e_/*N*_a_. Spectral correction (emission arm) was applied to the raw data after background subtraction, and from these spectrally corrected curves the quantum yield was calculated using aF900 software wizard.

**Scheme S1.** Synthetic procedures of **[tBCzDBFDP]_2_Cu_4_I_4_**.

*2. Synthesis Details*

**3,6-Di-*tert*-butyl-9-(dibenzo[b,d]furan-2-yl)-9H-carbazole:** In Ar, 2-Bromodibenzofuran (12.30 g, 50 mmol), 3,6-di-*tert*-butyl-9H-carbazole (27.9 g, 100 mmol), K_2_CO_3_ (13.8 g, 100 mmol) and copper powder (0.7 g, 11 mmol) were mixed in 200 mL of nitrobenzene and stirred for 48 h at 200 ^o^C. After the removal of the solvent, the mixture was extracted from water and dichloromethane (3 × 30 mL). The organic layer was combined and dried with anhydrous Na_2_SO_4_. The solvent was removed in vacuo. The residue was purified by flash column chromatography with the eluant of DCM:PE (1:10) to afford white powder (16 g) with a yield of 72%. ^1^H NMR (TMS, CDCl_3_, 400 MHz): *δ* = 8.176-8.173 (d, *J* = 1.2 Hz, 2H), 8.093-8.088 (d, *J* = 2 Hz, 1H), 7.934-7.915 (d, *J* = 7.6 Hz, 1H), 7.760-7.739 (d, *J* = 8.4 Hz, 1H), 7.652-7.587 (m, 2H), 7.537-7.517 (t, *J* = 8 Hz, 1H), 7.480-7.454 (dd, *J*_1_ = 1.6 Hz, *J*_2_ = 8.4 Hz, 2H), 7.390-7.353 (t, *J* = 7.6 Hz, 1H), 7.322-7.301 (d, *J* = 8.4 Hz, 2H), 1.477 ppm (s, 18H). ^13^C NMR (TMS, CDCl_3_, 100 MHz): *δ* = 150.990, 154.892, 142.793, 139.968, 133.149, 127.821, 126.388, 125.610, 123.911, 123.641, 123.230, 123.050, 120.919, 119.456, 116.284, 112.758, 111.958, 109.050, 34.767, 32.061 ppm. LDI-TOF: m/z (%) 445 (100) [M^+^]. Elemental Analysis for C_32_H_31_NO: C 86.25, H 7.01, N 3.14; found: C 86.27, H 7.03, N 3.18.

**9-(4,6-Bis(diphenylphosphanyl)dibenzo[b,d]furan-2-yl)-3,6-di-*tert*-butyl-9H-carbazole (tBCzDBFDP):** In Ar, 3,6-di-*tert*-butyl-9-(dibenzo[b,d]furan-2-yl)-9H-carbazole (3.3 g, 7.4 mmol) and N,N,N’,N’-tetramethylethylenediamine (TMEDA, 5.55 mL, 37 mmol) were dissolved in anhydrous diethyl ether (50 mL) and cooled to -78 ^o^C. Then, n-butyllithium (2.5 M in hexane, 14.8 mL, 37 mmol) was added dropwise under stir and further reacted for 16 h. Then, a solution of chlorodiphenylphosphine (7.4 mL, 40.7 mmol) in diethyl ether (10 mL) was added dropwise. The cold bath was removed, and the reaction mixture was stirred for another 16 h. After then, the reaction was quenched with water (50 mL). The mixture was extracted with dichloromethane (3 × 50 mL). The organic layer was combined and dried with anhydrous Na_2_SO_4_. The solvent was removed in vacuo. The residue was purified by flash column chromatography with the eluant of DCM:PE (1:10) to afford the ligand as white powder of 4.8 g with a yield of 80%. ^1^H NMR (TMS, CDCl_3_, 400 MHz): *δ* = 8.104-8.100 (d, *J* = 1.6 Hz, 2H), 8.054-8.049 (d, *J* = 2 Hz, 1H), 7.901-7.882 (d, *J* = 7.6 Hz, 1H), 7.415-7.388 (dd, *J*_1_ = 2 Hz, *J*_2_ = 8.8 Hz, 2H), 7.349-7.299 (m, 10H), 7.280-7.260 (m, 4H), 7.246-7.184 (m, 10H), 7.137-7.103 (m, 1H), 1.452 ppm (s, 18H). ^13^C NMR (TMS, CDCl_3_, 100 MHz): *δ* = 142.829, 139.530, 133.924, 133.774, 133.723, 133.572, 128.908, 128.780, 128.557, 128.482, 128.394, 123.562, 123.210, 121.674, 119.559, 116.177, 109.001, 67.988, 34.726, 32.017, 25.627 ppm. ^31^P NMR (TMS, CDCl_3_, 162 MHz): *δ* = -16.277, -17.019 ppm. LDI-TOF: m/z (%) 813 (100) [M^+^]. Elemental Analysis for C_56_H_49_NOP_2_: C 82.63, H 6.07, N 1.72; found: C 82.64, H 6.06, N 1.75.

**[tBCzDBFDP]_2_Cu_4_I_4_:** 1 mmol of tBCzDBFDP (813 mg) and 2 mmol of CuI (380 mg) were dissolved in 20 mL of CH_2_Cl_2_. The mixture was stirred for 4 h. Then, the solvent was evaporated to obtain crude complex, which was further recrystallization from CH_2_Cl_2_/ether solution to afford 1.05 g of yellow crystal with a yield of 88%. ^1^H NMR (TMS, CDCl_3_, 400 MHz): *δ* = 8.193 (s, 2H), 8.090 (s, 4H), 8.022-8.003 (d, *J* = 7.6 Hz, 2H), 7.673-7.656 (m, 16H), 7.532-7.517 (m, 2H), 7.369-7.322 (m, 32H), 7.044-7.022 (d, *J* = 8.8 Hz, 4H), 1.450 ppm (s, 36H). ^13^C NMR (TMS, CDCl_3_, 100 MHz): *δ* = 143.041, 139.056, 134.424, 134.284, 134.120, 133.493, 129.561, 128.435, 128.343, 128.277, 128.185, 123.681, 123.356, 116.182, 108.899, 34.723, 31.996, 25.625, 1.029, 0.004 ppm. ^31^P NMR (TMS, CDCl_3_, 162 MHz): *δ* = -29.897, -30.646 ppm. LDI-TOF: m/z (%) 2390 (100) [M^+^]. Elemental Analysis for C_112_H_98_N_2_O_2_P_4_Cu_4_I_4_: C 56.29, H 4.13, N 1.17; found: C 56.32, H 4.11, N 1.21.

**Scheme S2.** Synthetic procedures of **[PTZDBFDP]_2_Cu_4_I_4_**.

**10-(Dibenzo[b,d]furan-2-yl)-10H-phenothiazine:** In Ar, 2-bromodibenzofuran (12.30 g, 50 mmol), 10H-phenothiazine (10.95 g, 55 mmol), Palladiumacetate (0.30 g, 2.5 mmol), t-BuOK (6.16 g, 55 mmol) and (t-Bu)_3_P (9.50 ml, 3.8 mmol) were mixed in 300 mL of Xylenes and stirred for 12 h at 130 ^o^C. After the removal of the solvent, the mixture was extracted from water and dichloromethane (3 × 30 mL). The organic layer was combined and dried with anhydrous Na_2_SO_4_. The solvent was removed in vacuo. The residue was purified by flash column chromatography with the eluant of DCM:PE (1:15) to afford white powder (15.5 g) with a yield of 85%. ^1^H NMR (TMS, CDCl_3_, 400 MHz): *δ* = 8.004 (s, 1H), 7.929-7.910 (d, *J* = 7.6 Hz, 1H), 7.796-7.775 (d, *J* = 8.4 Hz, 1H), 7.640-7.619 (d, *J* = 8.4 Hz, 1H), 7.529-7.460 (m, 2H), 7.385-7.348 (t, *J* = 7.6 Hz, 1H), 7.029-7.007 (m, 2H), 6.803 (s, 4H), 6.203-6.187 ppm (m, 2H). ^13^C NMR (TMS, CDCl_3_, 100 MHz): *δ* = 156.899, 155.410, 127.930, 126.857, 126.813, 126.754, 126.662, 123.845, 123.172, 122.549, 122.477, 122.443, 120.995, 115.815, 113.793, 111.985 ppm. LDI-TOF: m/z (%) 365 (100) [M^+^]. Elemental Analysis for C_24_H_15_NOS: C 78.88, H 4.14, N 3.83; found: C 78.90, H 4.17, N 3.87.

**10-(4,6-bis(diphenylphosphanyl)dibenzo[b,d]furan-2-yl)-10H-phenothiazine (PTZDBFDP):** In Ar, 10-(dibenzo[b,d]furan-2-yl)-10H-phenothiazine (2.70 g, 7.4 mmol) and N,N,N’,N’-tetramethylethylenediamine (TMEDA, 5.55 mL, 37 mmol) were dissolved in anhydrous diethyl ether (50 mL) and cooled to -78 ^o^C. Then, n-butyllithium (2.5 M in hexane, 14.8 mL, 37 mmol) was added dropwise under stir and further reacted for 16 h. Then, a solution of chlorodiphenylphosphine (7.4 mL, 40.7 mmol) in diethyl ether (10 mL) was added dropwise. The cold bath was removed, and the reaction mixture was stirred for another 16 h. After then, the reaction was quenched with water (50 mL). The mixture was extracted with dichloromethane (3 × 50 mL). The organic layer was combined and dried with anhydrous Na_2_SO_4_. The solvent was removed in vacuo. The residue was purified by flash column chromatography with the eluant of DCM:PE (1:10) to afford the ligand as white powder of 3.25 g with a yield of 60%. ^1^H NMR (TMS, CDCl_3_, 400 MHz): *δ* = 7.955-7.950 (d, *J* = 2 Hz, 1H), 7.881-7.862 (d, *J* = 7.6 Hz, 1H), 7.336-7.267 (m, 15H), 7.248-7.217 (m, 6H), 7.164-7.145 (m, 1H), 7.114-7.080 (m, 1H), 6.995-6.972 (m, 2H), 6.800-6.776 (m, 4H), 6.117-6.093 ppm (m, 2H). ^13^C NMR (TMS, CDCl_3_, 100 MHz): *δ* = 143.991, 136.937, 136.814, 132.768, 132.688, 132.590, 132.253, 132.154, 132.020, 131.905, 131.794, 128.933, 128.833, 128.745, 128.628, 128.536, 127.522, 127.012, 123.794, 123.033, 121.090, 116.491, 67.981, 26.922, 25.618, 1.026, 0.913, 0.000 ppm. ^31^P NMR (TMS, CDCl_3_, 162 MHz): *δ* = -16.523, -16.834 ppm. LDI-TOF: m/z (%) 733 (100) [M^+^]. Elemental Analysis for C_48_H_33_NOP_2_S: C 78.57, H 4.53, N 1.91; found: C 78.60, H 4.55, N 1.94.

**[PTZDBFDP]_2_Cu_4_I_4_:** In Ar, 1 mmol of PTZDBFDP (733 mg) and 2 mmol of CuI (380 mg) were dissolved in 20 mL of CH_2_Cl_2_. The mixture was stirred for 4 h. Then, the solvent was evaporated to obtain crude complex, which was further recrystallization from CH_2_Cl_2_/ether solution to afford 0.98 g of yellow crystal with a yield of 88%. ^1^H NMR (TMS, CDCl_3_, 400 MHz): *δ* = 8.052 (s, 2H), 7.987-7.967 (m, 2H), 7.684-7.594 (m, 16H), 7.358-7.304 (m, 30H), 7.001-6.983 (m, 4H), 6.799-6.717 (m, 8H), 5.988-5.968 ppm (d, *J* = 8 Hz, 4H). ^13^C NMR (TMS, CDCl_3_, 100 MHz): *δ* = 159.416, 159.275, 158.073, 157.919, 144.468, 138.142, 135.968, 135.941, 135.428, 134.385, 134.248, 134.125, 133.988, 129.651, 129.568, 128.366, 128.277, 128.188, 126.843, 126.799, 125.165, 123.583, 123.194, 122.612, 120.185, 118.114, 115.886, 58.506, 18.459, 1.031, 0.006 ppm. ^31^P NMR (TMS, CDCl_3_, 162 MHz): *δ* = -29.926, -31.200 ppm. LDI-TOF: m/z (%) 2228 (100) [M^+^]. Elemental Analysis for C_96_H_66_N_2_O_2_P_4_S_2_Cu_4_I_4_: C 51.72, H 2.98, N 1.26; found: C 51.75, H 2.99, N 1.29.

*3. DFT and TDDFT Calculation*

DFT computations were carried out with different parameters for structure optimizations and vibration analyses. The ground, singlet and triplet states of clusters in vacuum were optimized by the restricted and unrestricted formalism of Beck's three-parameter hybrid exchange functional(*1*) and Lee, and Yang and Parr correlation functional(*2*) B3LYP/6-31G(d,p), respectively. The fully optimized stationary points were further characterized by harmonic vibrational frequency analysis to ensure that real local minima had been found without imaginary vibrational frequency. The total energies were also corrected by zero-point energy both for the ground state and triplet state. Natural transition orbital (NTO) analysis was performed on the basis of optimized ground-state geometries at the level of B3LYP/6-31G(d,p).(*3*) The contours were visualized with Gaussview 5.0. All computations were performed using the Gaussian 09 package.(*4*)

*4. Device Fabrication and Testing*

The devices configuration was ITO|PEDOT:PSS (40 nm)|BCPO:**Cu_4_I_4_** cluster (*x*%wt., 40 nm)|DPEPO (10 nm)|TmPyPB (50 nm)|LiF (1 nm)|Al (100 nm), in which poly(3,4-ethylenedioxythiophene): poly(styrenesulfonate) (PEDOT:PSS) and LiF are hole and electron injecting layers; while BCPO, DPEPO and TmPyPB served as host, exciton blocking and electron transporting layers, respectively. PEDOT:PSS layer was spin-coated on the patterned ITO-coated glass substrate after oxygen plasma treatment. To remove the residual water trapped in the film, PEDOT:PSS layer was baked at 120 ^o^C for 20 minutes in the glove-box. The emitting layer was spin-coated from chlorobenzene at a concentration of 10 mg/ml. After the spin-coating, the sample was transferred to a high vacuum evaporation system. The last layers were deposited through a shadow mask in the chamber under a base pressure of ~10^-4^ Pa. Finally, all the devices were encapsulated with UV epoxy resin in the glove-box before carrying out the luminance-current-voltage measurement. The emission intensity was measured with a calibrated Si photodiode. The external quantum efficiency of the device was calculated with the assumption of a Lambertian distribution. The electroluminescent spectrum was determined by a calibrated PR655 spectrometer. Devices of each cluster were repeated for 12 times to investigate the repeatability. It is showed that the variations of EQE were within 1% (**Figure S13**). The data reported were from the devices mostly close to the average values.

### Thermal Properties


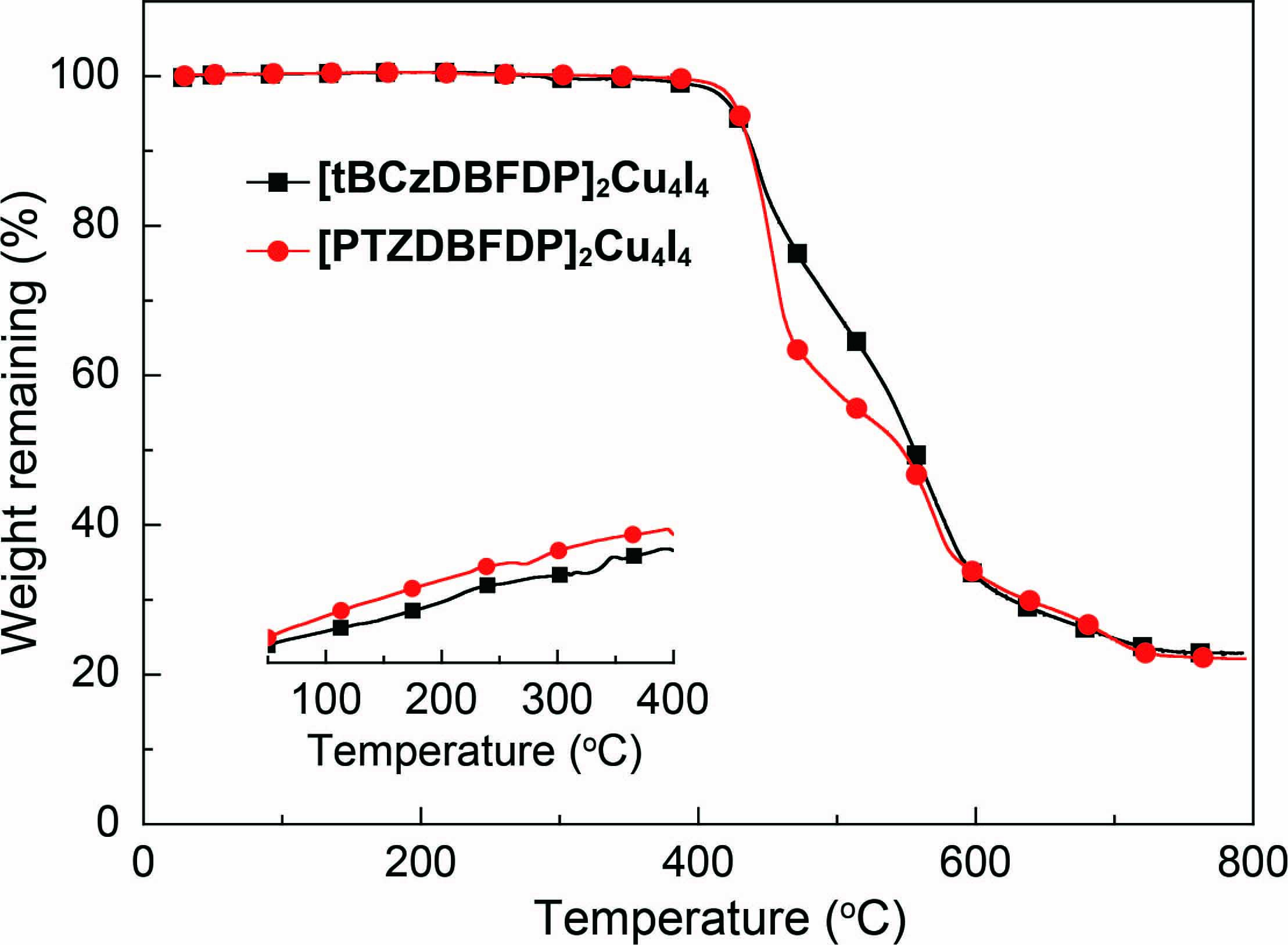


**Figure S1.** TGA and DSC curves of **[tBCzDBFDP]_2_Cu_4_I_4_** and **[PTZDBFDP]_2_Cu_4_I_4_**.

### Morphological Properties


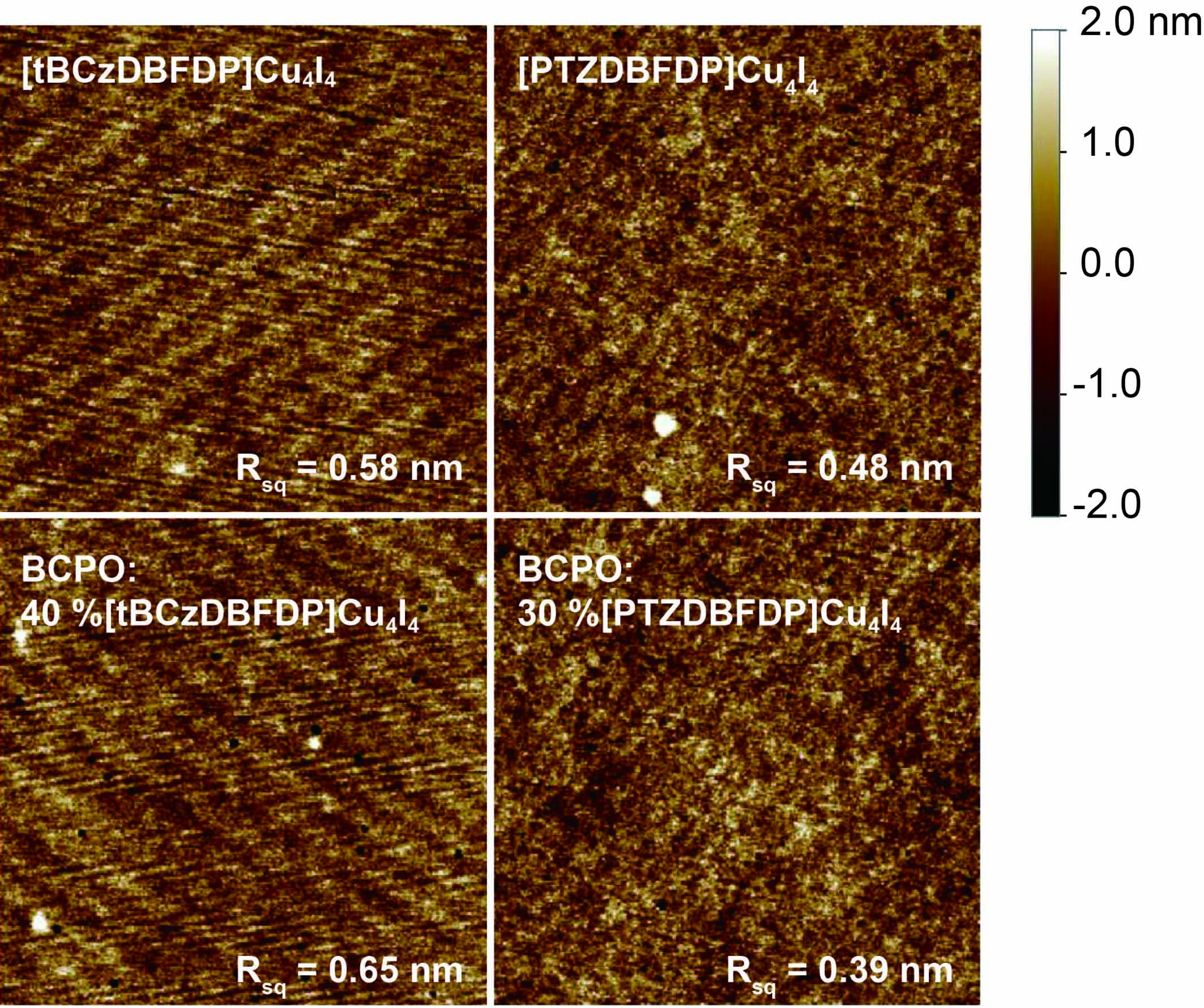


**Figure S2.** Atom force microscopy (AFM) images of neat and BCPO hosted films based on **[tBCzDBFDP]_2_Cu_4_I_4_** and **[PTZDBFDP]_2_Cu_4_I_4_** by spin coating.

### DFT Simulation


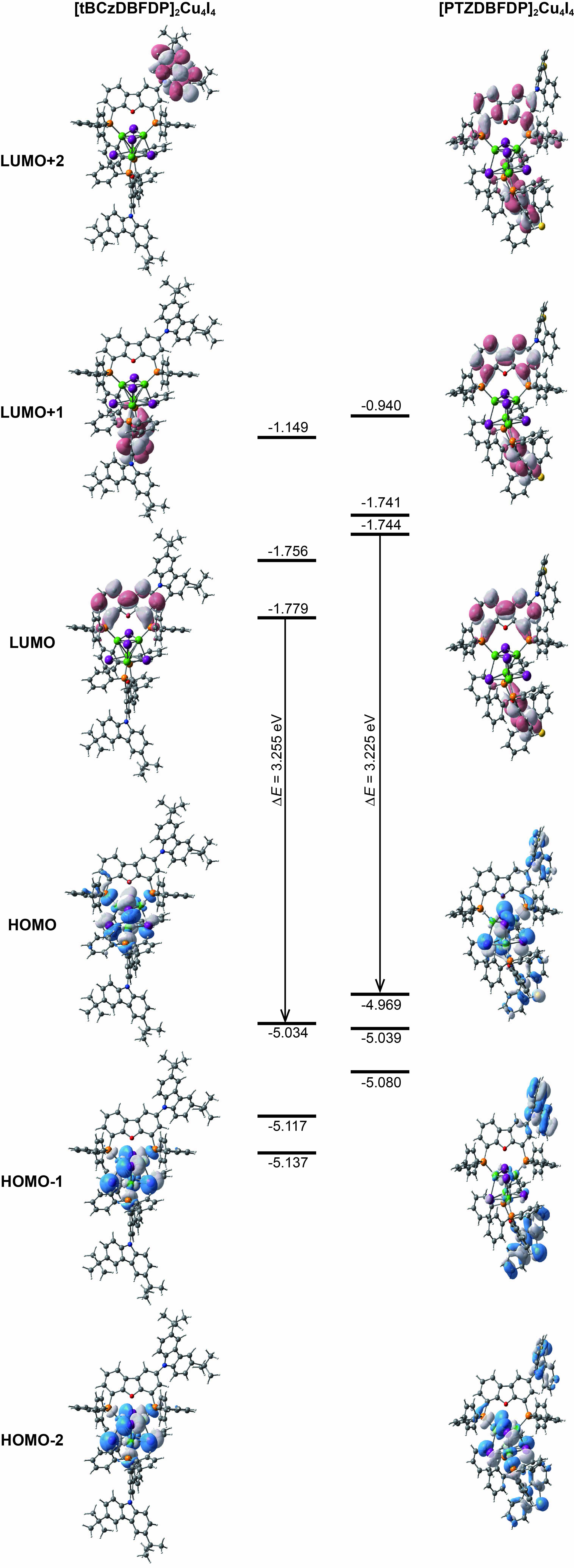


**Figure S3.** FMO energy levels and contours of **[tBCzDBFDP]_2_Cu_4_I_4_** and **[PTZDBFDP]_2_Cu_4_I_4_** simulated with B3LYP/6-31G* method.

### Electrochemical Properties


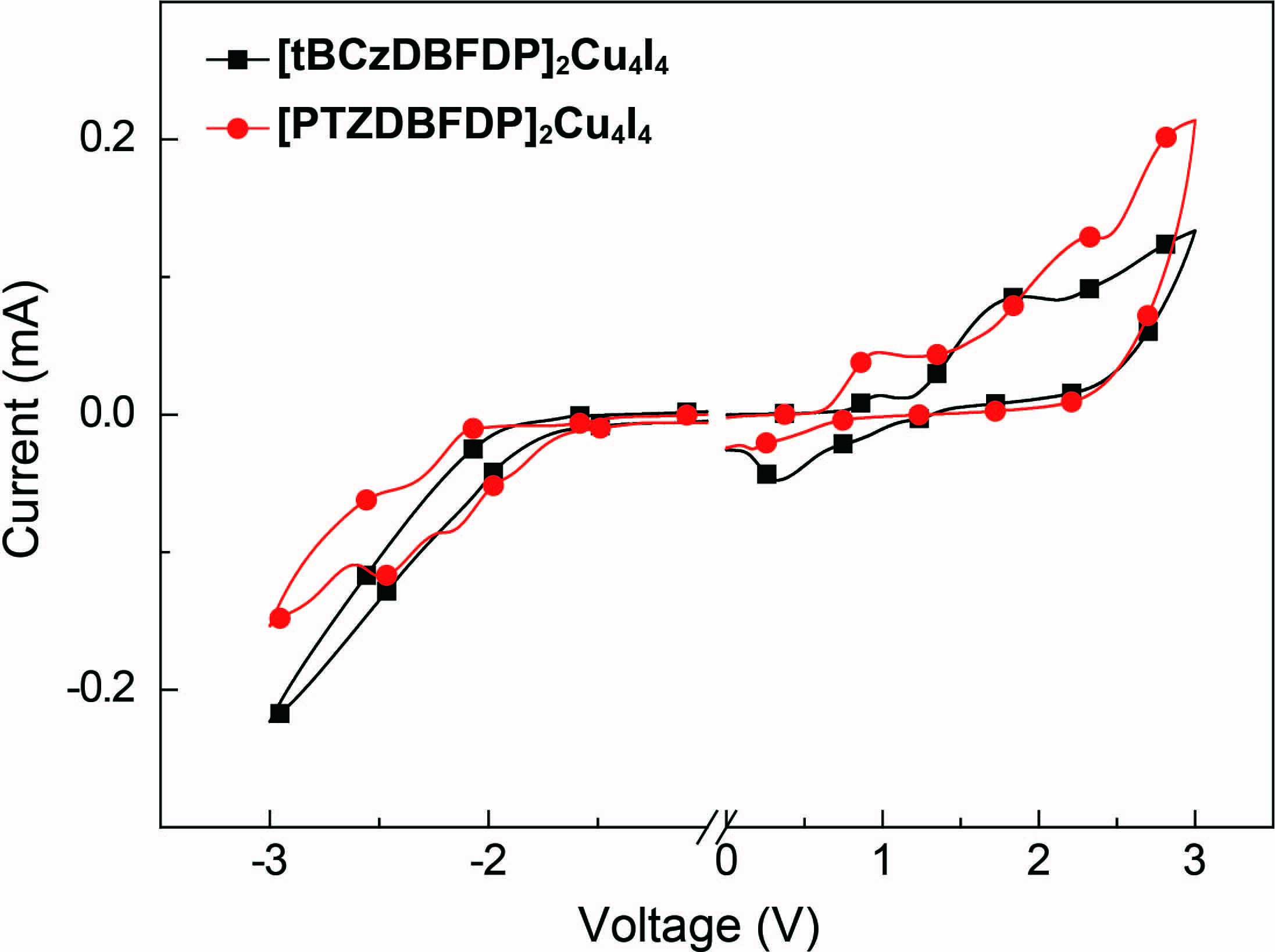


**Figure S4.** CV curves of **[tBCzDBFDP]_2_Cu_4_I_4_** and **[PTZDBFDP]_2_Cu_4_I_4_** measured at room temperature with the scanning rate of 100 mV s^-1^.

### Photophysical Properties


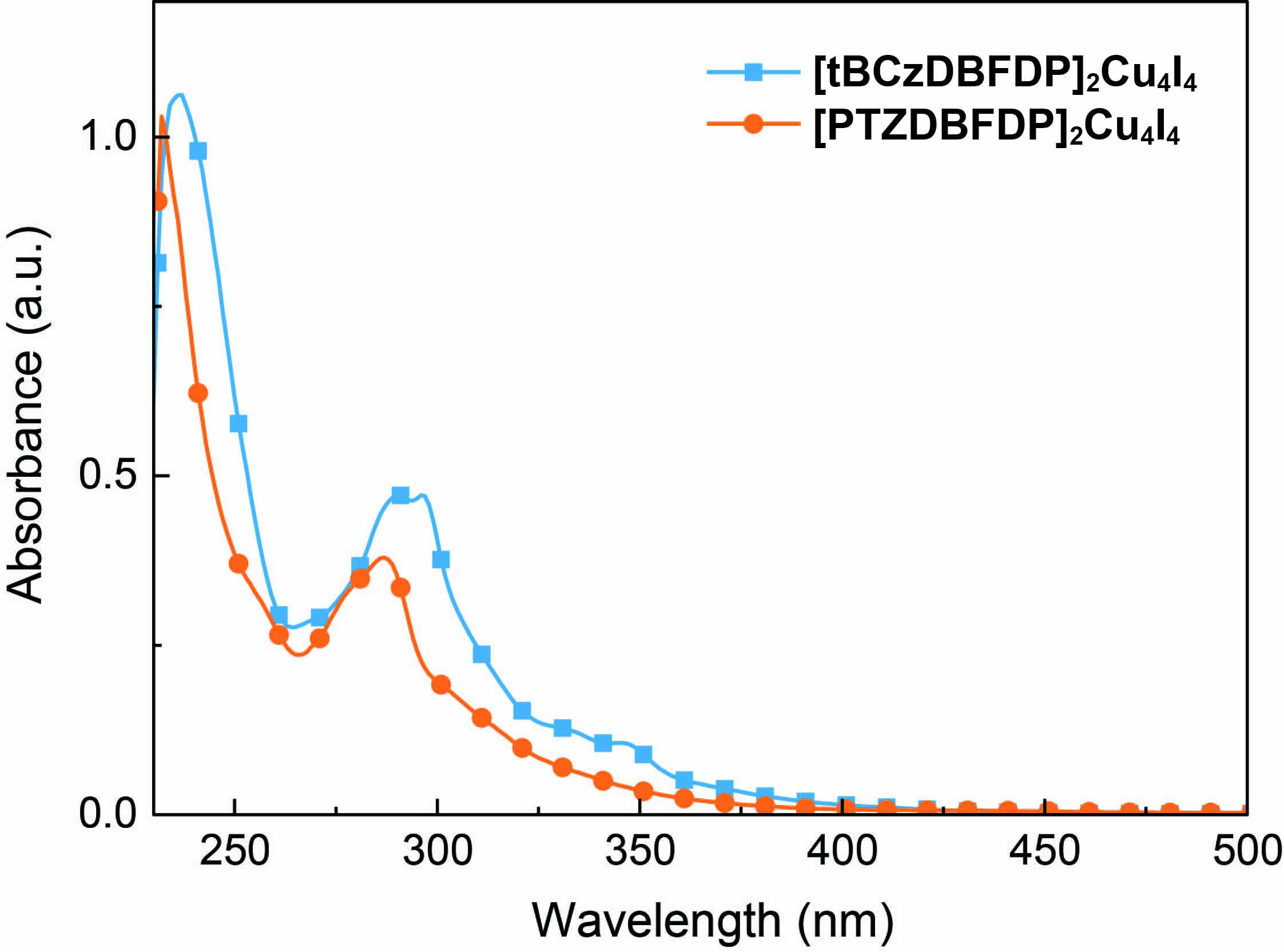


**Figure S5.** Electronic absorption spectra of **[tBCzDBFDP]_2_Cu_4_I_4_** and **[PTZDBFDP]_2_Cu_4_I_4_** in dilute dichloromethane (10^-6^ mol L^-1^).


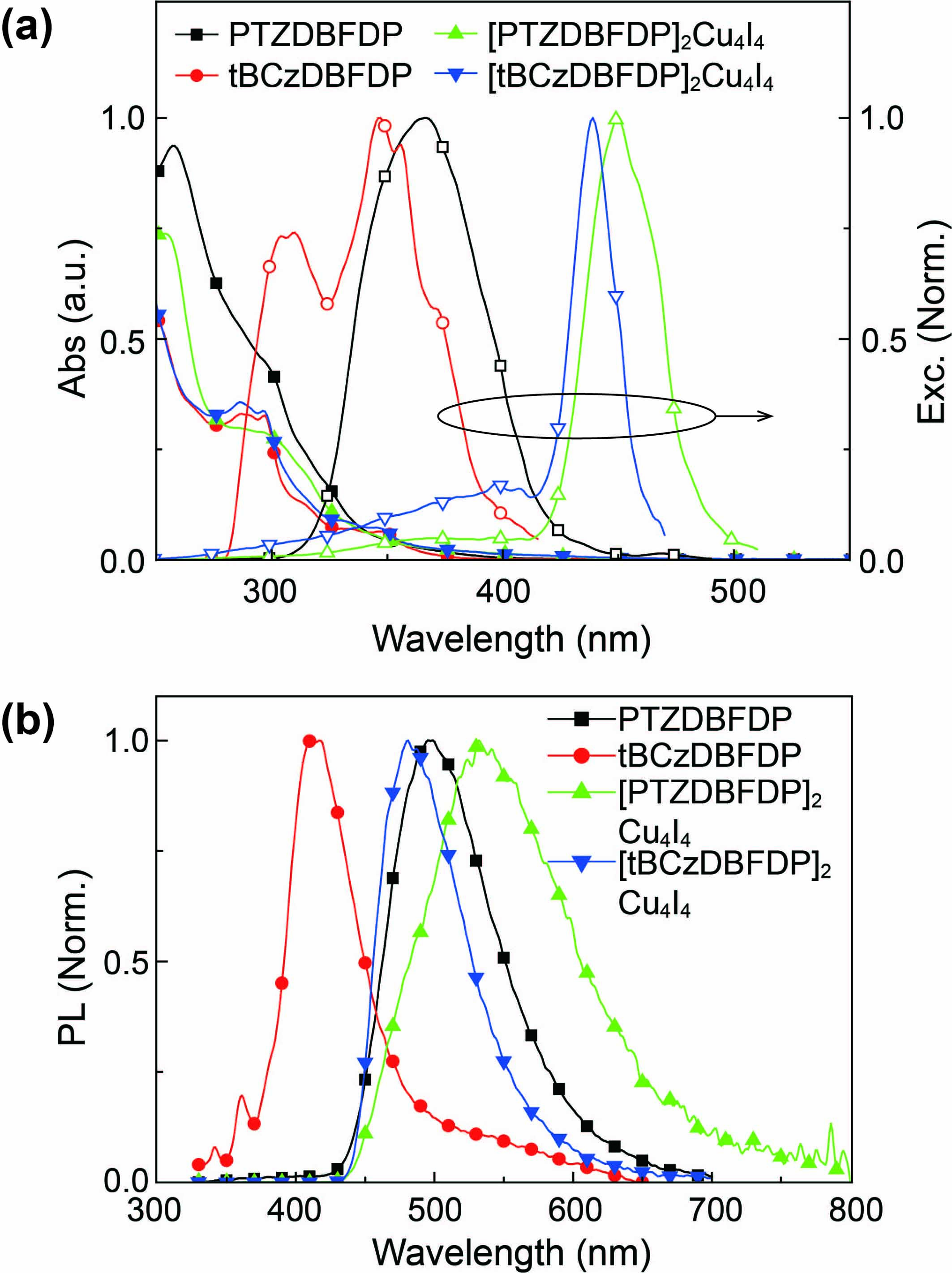


**Figure S6.** Electronic absorption, excitation (a) and PL (b) spectra of tBCzDBFDP, PTZDBFDP, **[tBCzDBFDP]_2_Cu_4_I_4_** and **[PTZDBFDP]_2_Cu_4_I_4_** in dilute toluene.


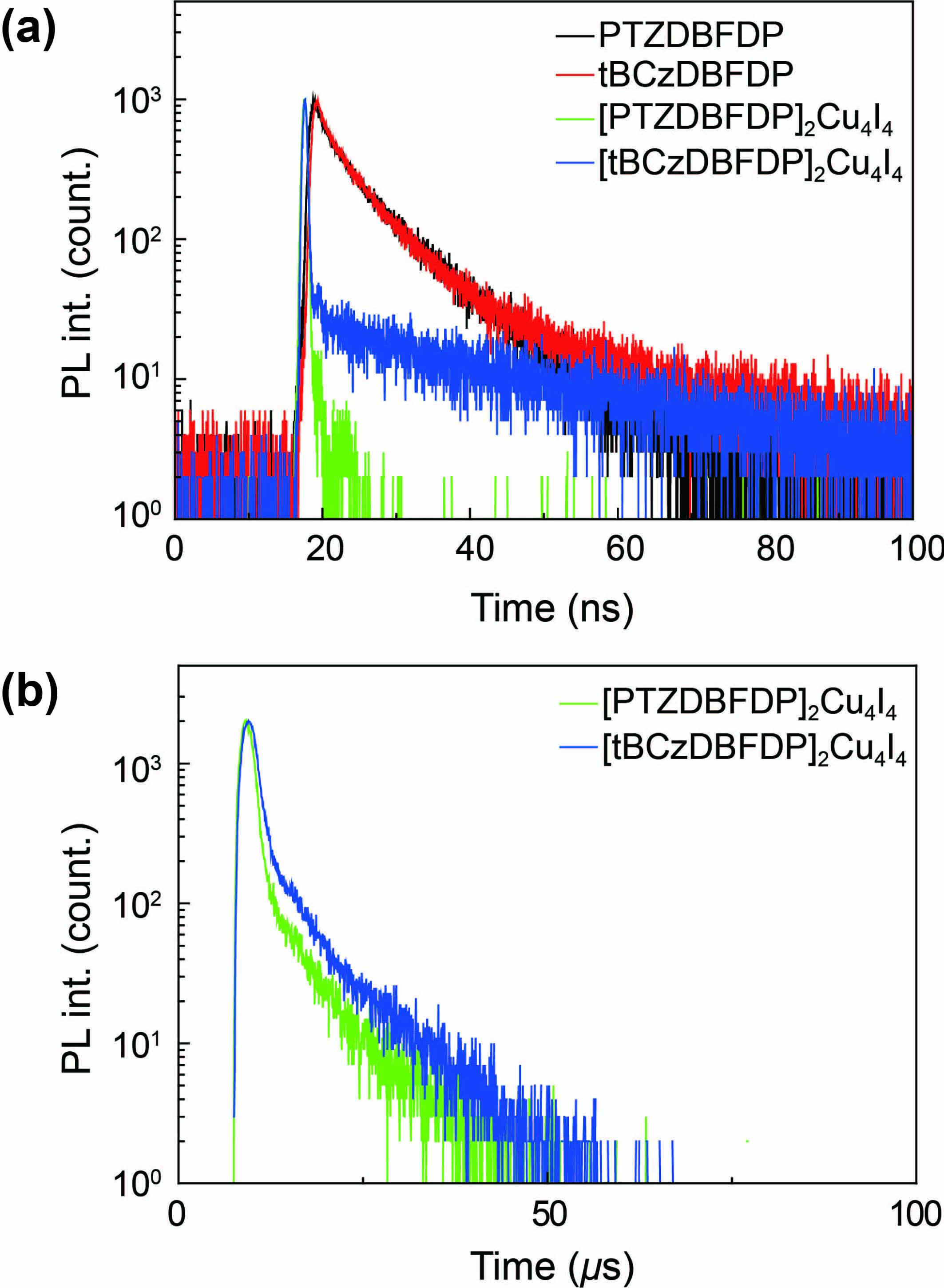


**Figure S7.** Time decays of tBCzDBFDP, PTZDBFDP, **tBCzDBFDP]_2_Cu_4_I_4_** and **[PTZDBFDP]_2_Cu_4_I_4_** at nanosecond (a) and microsecond (b) scales in dilute toluene.


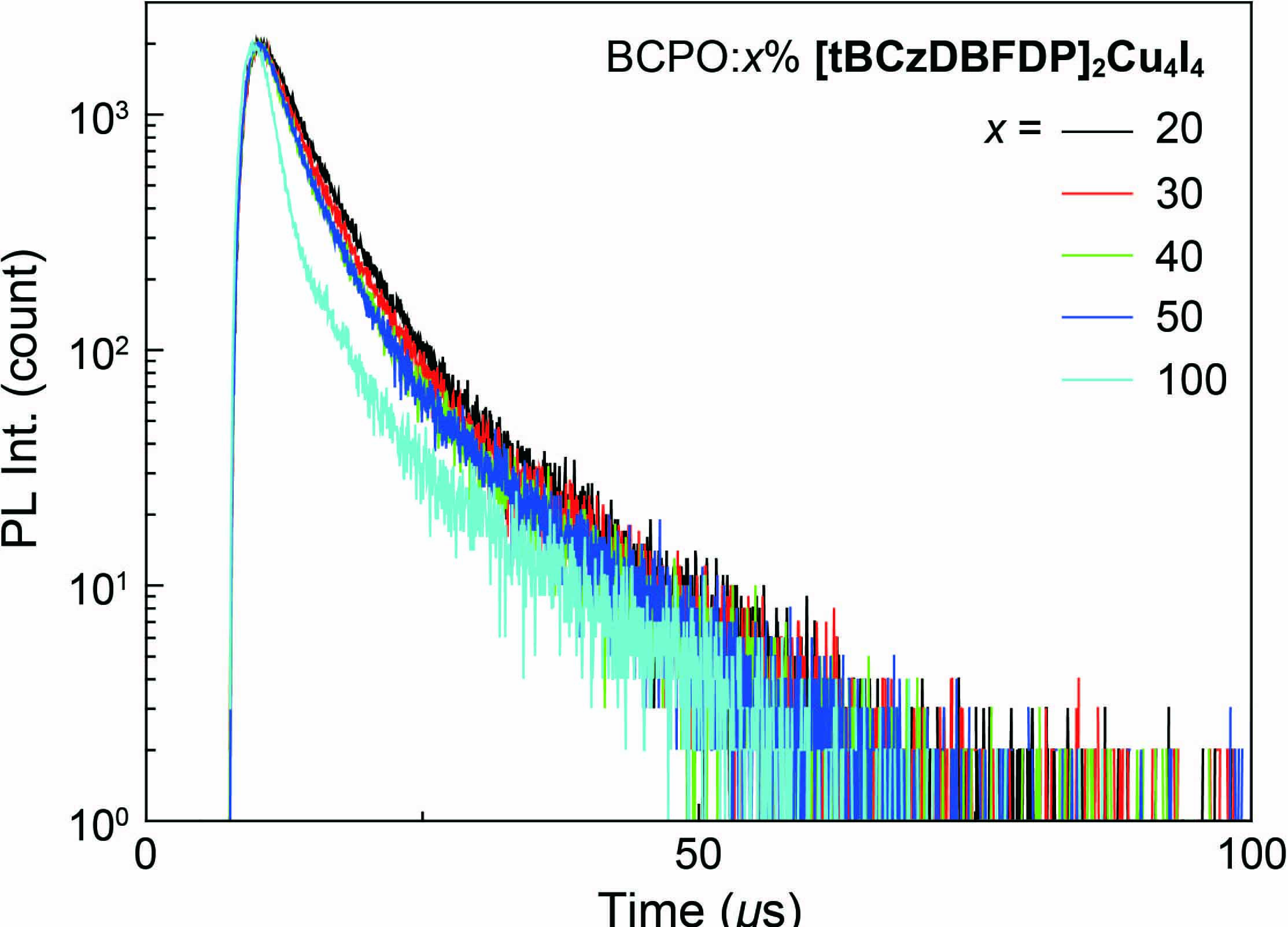


**Figure S8.** Doping concentration dependence of time decay curves for BCPO:*x*% **[tBCzDBFDP]_2_Cu_4_I_4_** films (*x* = 20-50 and 100).


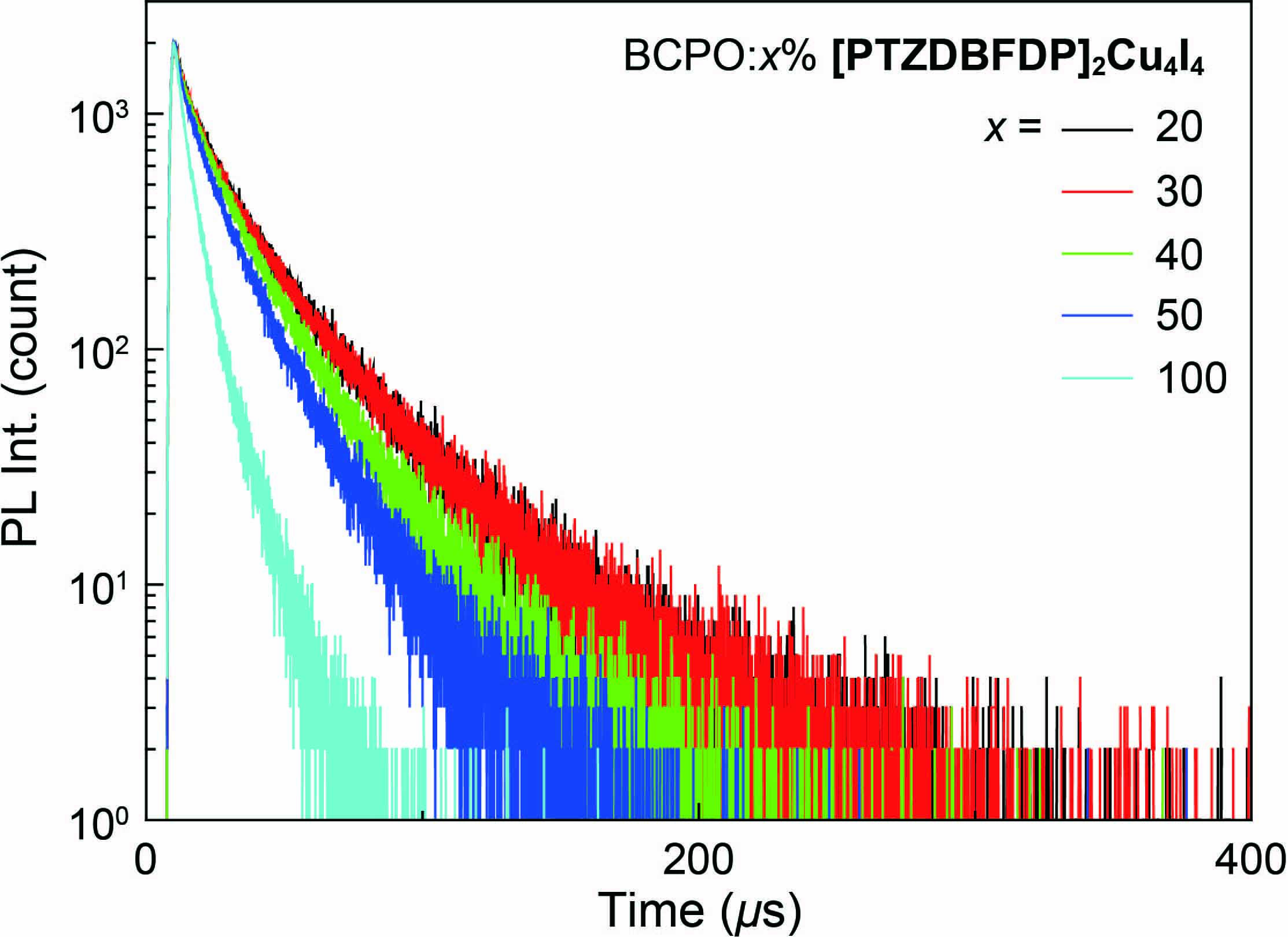


**Figure S9.** Doping concentration dependence of time decay curves for BCPO:*x*% **[PTZDBFDP]_2_Cu_4_I_4_** films (*x* = 20-50 and 100).


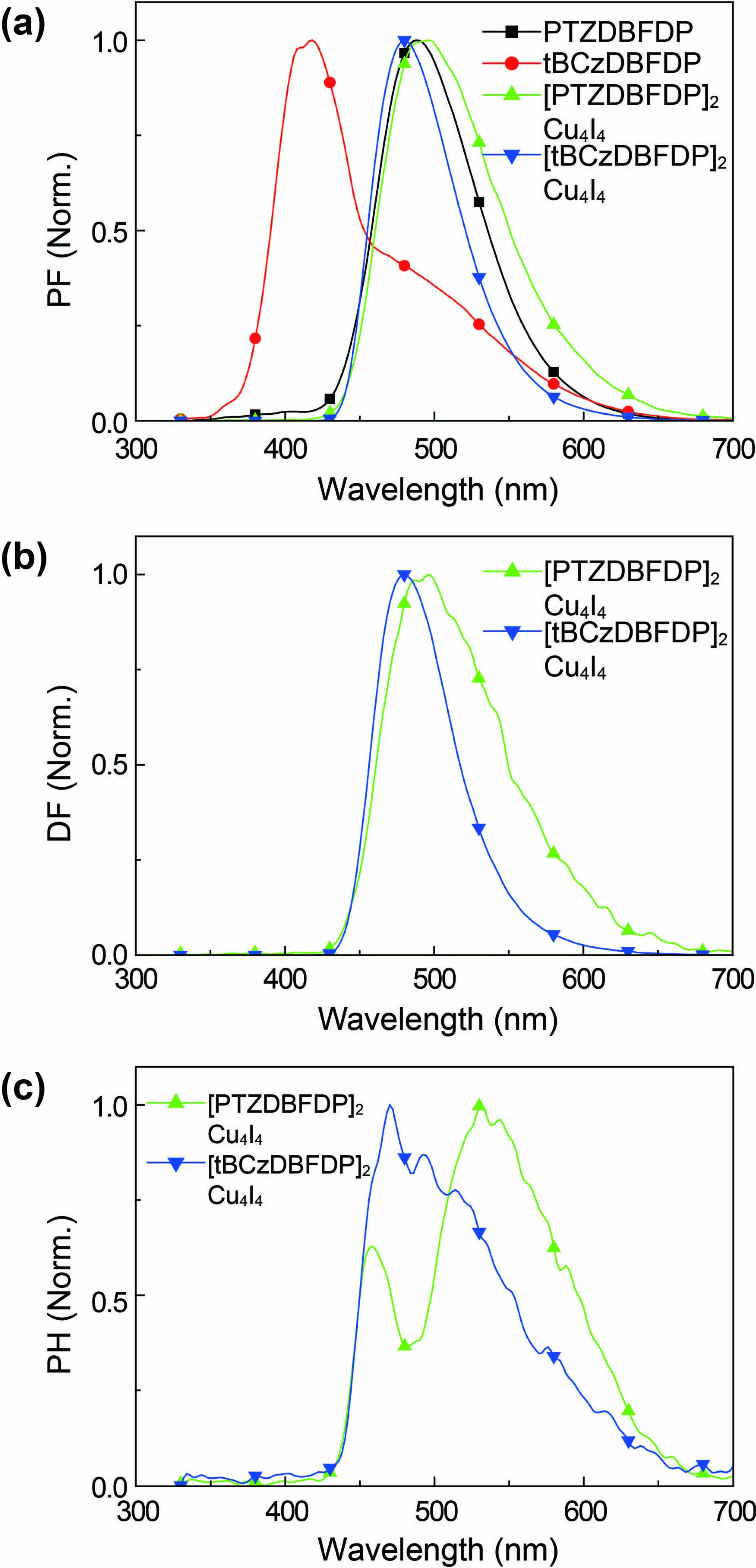


**Figure S10.** Prompt fluorescence (PF) spectra of tBCzDBFDP, PTZDBFDP, **[tBCzDBFDP]_2_Cu_4_I_4_** and **[PTZDBFDP]_2_Cu_4_I_4_** in dilute toluene.


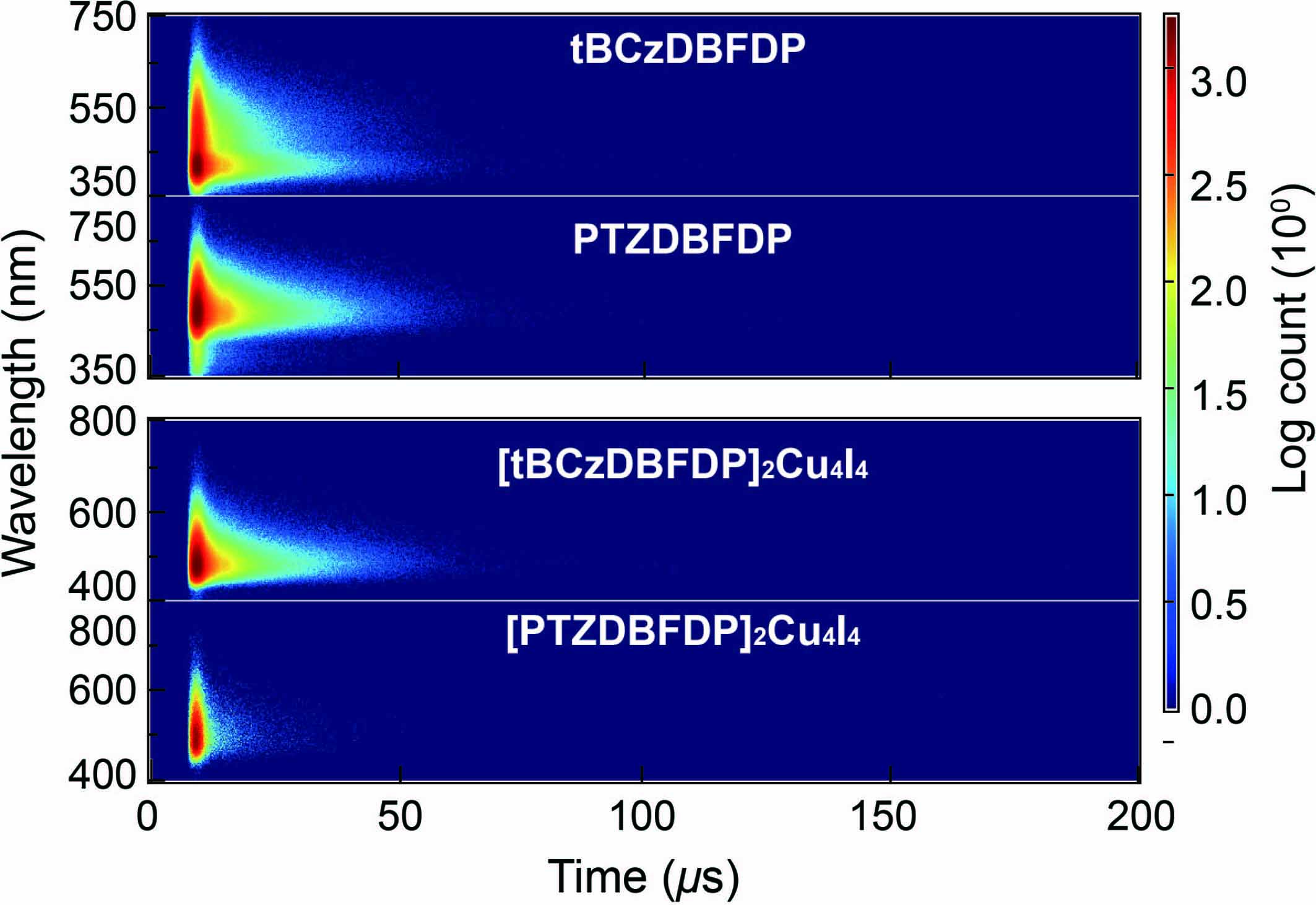


**Figure S11.** Time-resolved emission spectra of tBCzDBFDP, PTZDBFDP, **[tBCzDBFDP]_2_Cu_4_I_4_** and **[PTZDBFDP]_2_Cu_4_I_4_** in dilute toluene.


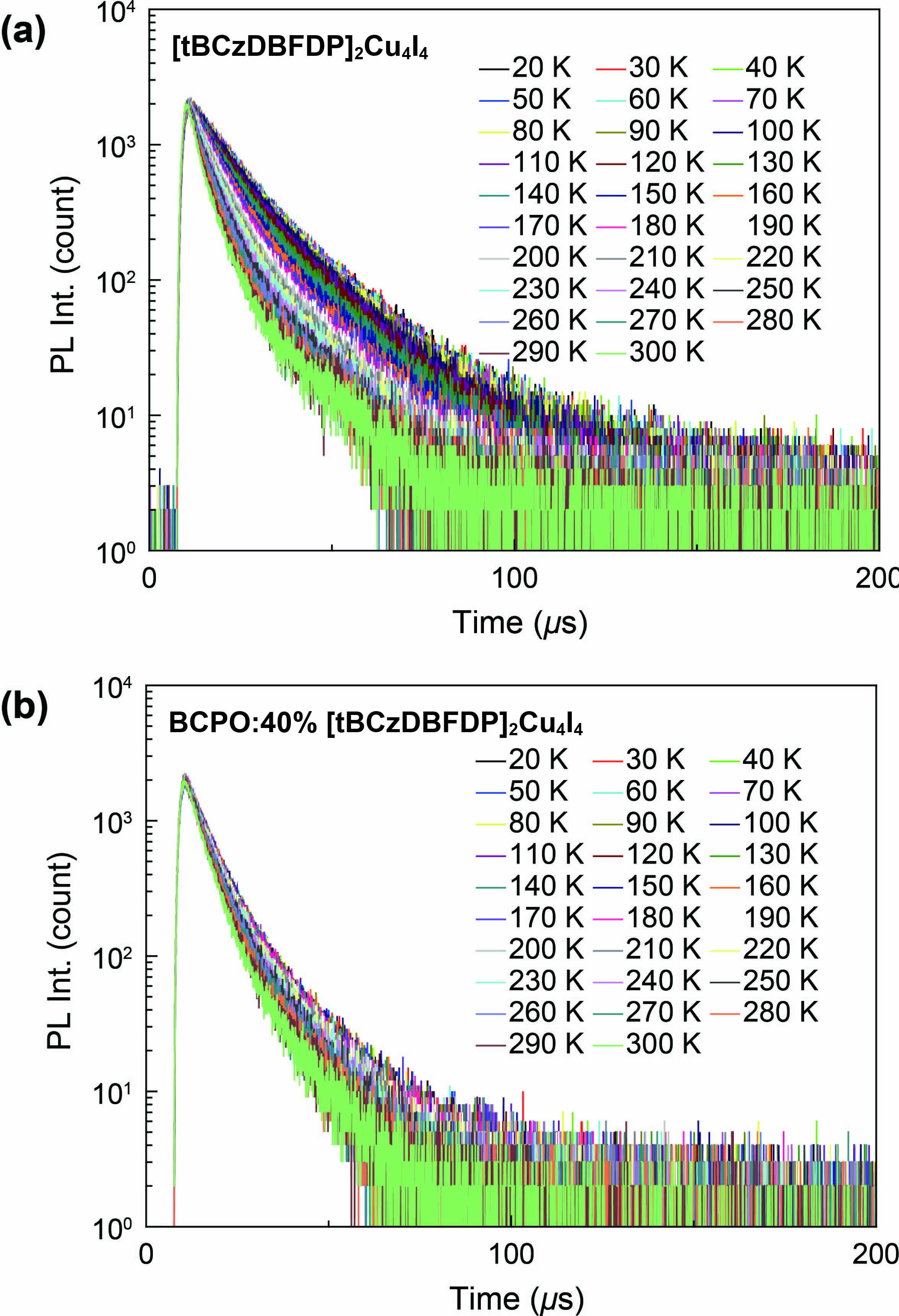


**Figure S8.** Time decay curves of BCPO:*x*% **[tBCzDBFDP]_2_Cu_4_I_4_** (*x* = 40 and 100 for neat film) in the temperature range from 20 to 300 K with an interval of 10 K.


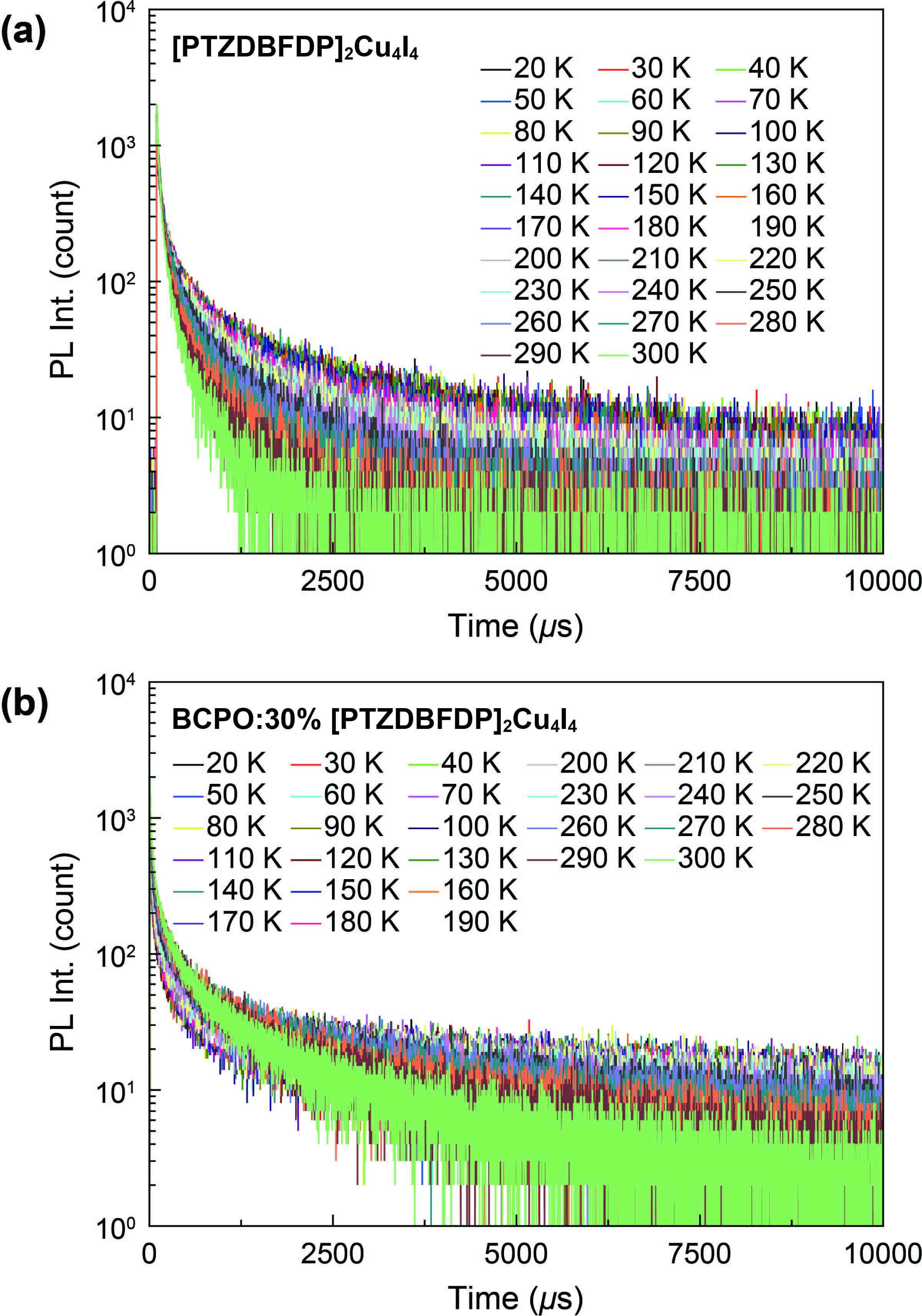


**Figure S9.** Time decay curves of BCPO:*x*% **[PTZDBFDP]_2_Cu_4_I_4_** (*x* = 30 and 100 for neat film) in the temperature range from 20 to 300 K with an interval of 10 K.


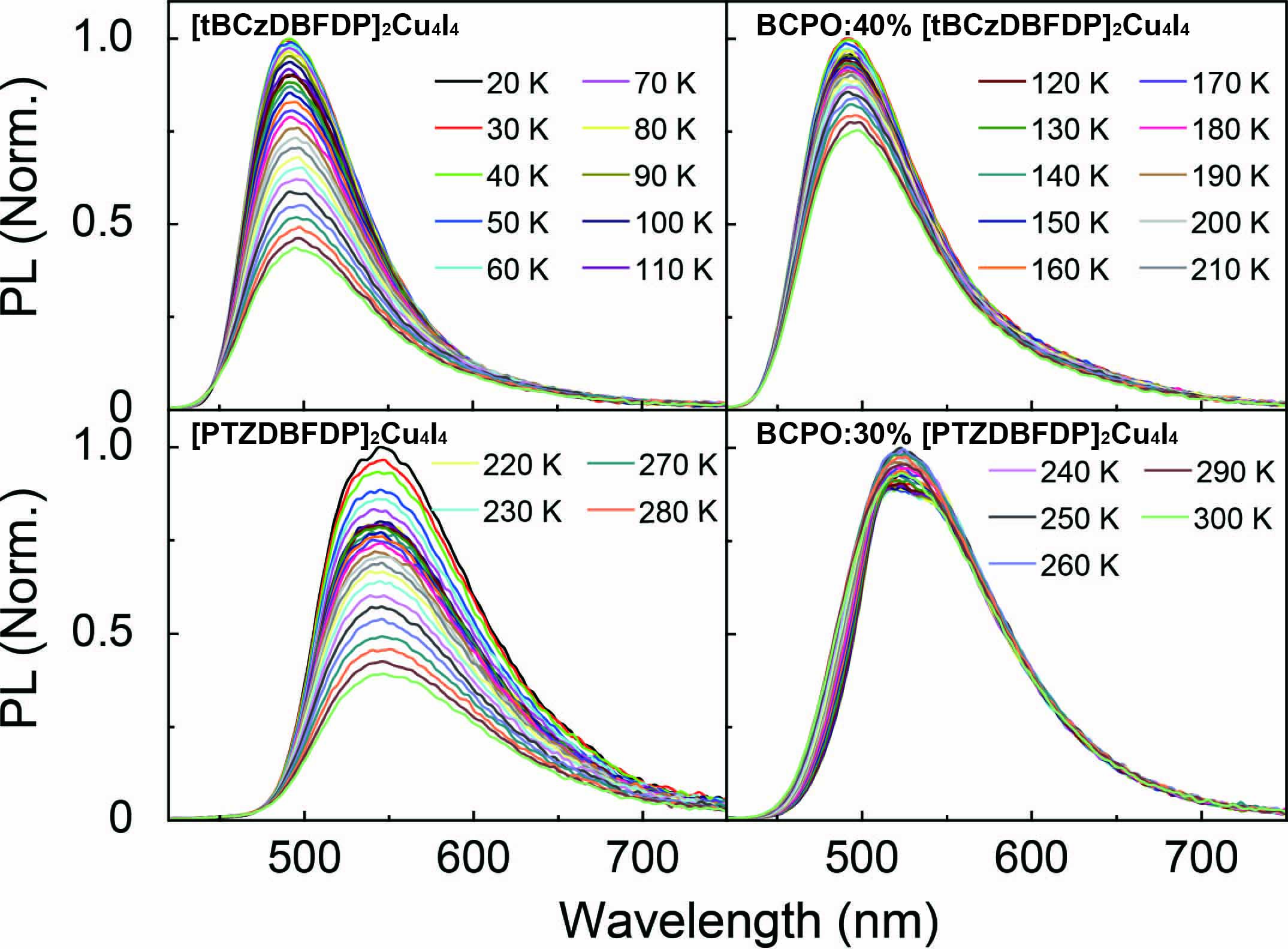


**Figure S10.** PL spectra of BCPO:*x*% **[tBCzDBFDP]_2_Cu_4_I_4_** and BCPO:*x*% **[PTZDBFDP]_2_Cu_4_I_4_** (*x* = 40 for the former, 30 for the latter, and 100 for neat films) in the temperature range from 20 to 300 K with an interval of 10 K.

### Table S1. Physical properties of the clusters.

| Cluster | *λ*_Abs._ (nm) | *λ*_PL_  (nm) | S_1_  (eV) | T_1_  (eV) | Δ*E*_ST_^[g]^  (eV) | *f*_S_ | *T*_g_ */ T*_m_ */ T*_d_  (^o^C) | HOMO  (eV) | LUMO  (eV) |
| --- | --- | --- | --- | --- | --- | --- | --- | --- | --- |
| **[tBCzDBFDP]_2_Cu_4_I_4_** | 237, 294, 347^[a]^  231, 247, 299, 347, 376^[b]^ | 495^[b]^  498^[b][c]^ | 2.77^[e]^  2.55^[d]^ | 2.70^[e]^  2.50^[f]^ | 0.07^[e]^  0.05^[d][f]^ | 0.014^[e]^ | 294, -, 427^[h]^ | -5.03^[e]^  -5.59^[i]^ | -1.78^[e]^  -3.15^[i]^ |
| **[PTZDBFDP]_2_Cu_4_I_4_** | 232, 287, 323^[a]^  220, 258, 305, 388^[b]^ | 547^[b]^ 523^[b][c]^ | 2.72^[e]^  2.38^[d]^ | 2.65^[e]^  2.34^[f]^ | 0.07^[e]^  0.04^[d][f]^ | 0.004^[e]^ | 313, -, 429^[h]^ | -4.97^[e]^  -5.42^[i]^ | -1.74^[e]^  -3.34^[i]^ |

[a] In DCM solution (10^-6^ mol L^-1^); [b] in film; [c] emission peaks of 30-40% doped BCPO films; [d] estimated according to 0-0 transition of prompt fluorescence; [e] Gaussian simulation results of single molecules; [f] estimated with 0-0 transition of phosphorescence; [g] singlet-triplet splitting; [h] temperature at weight loss of 5%; [i] calculated according to cyclic voltammetric results with Lippert-Mataga relationship.

### EL Performance


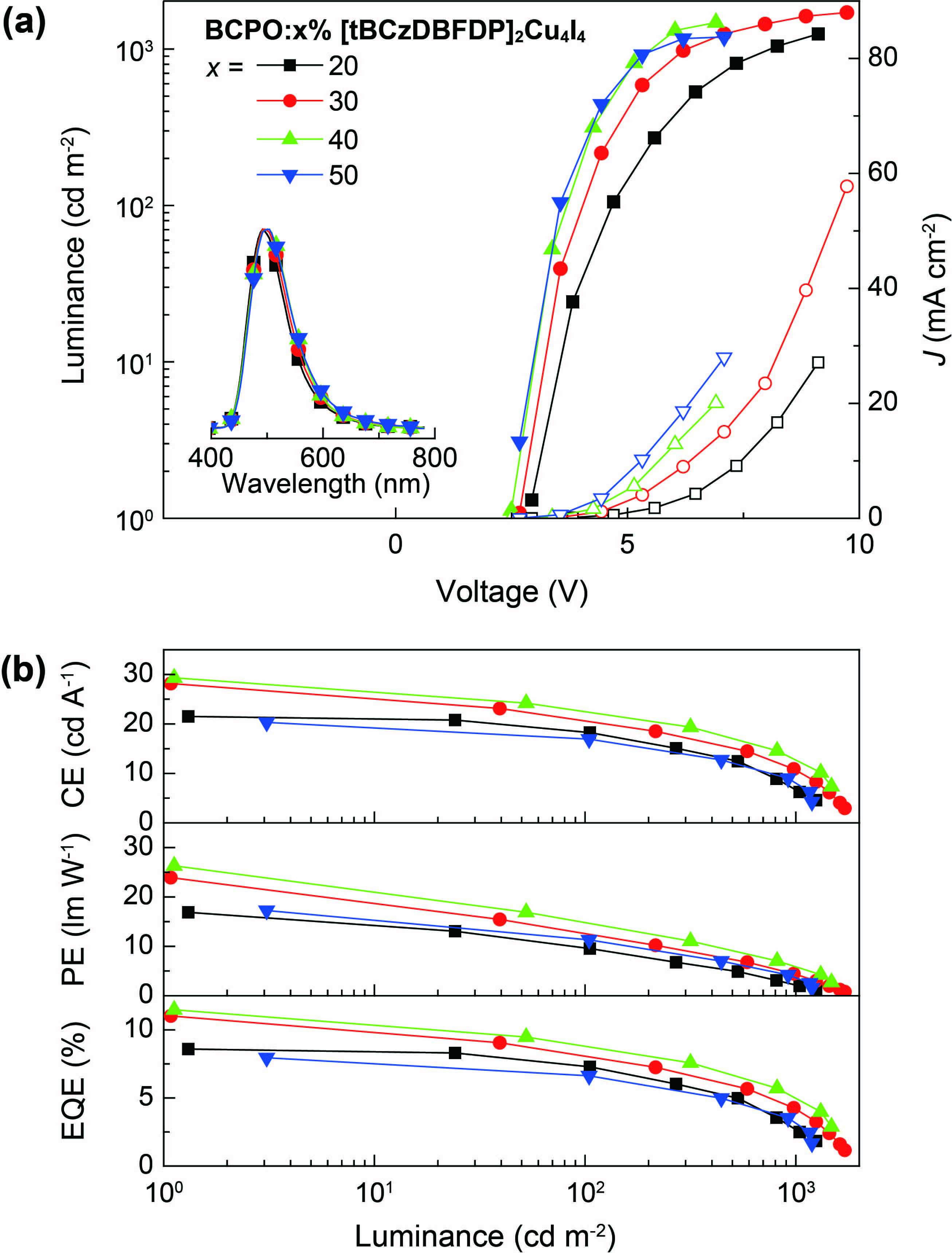


**Figure S11.** (**a**) EL spectra (inset) and Current density (*J*)-Voltage-Luminance characteristics of BCPO:*x*% **[tBCzDBFDP]_2_Cu_4_I_4_** based CLEDs at different *x*; (**b**) Efficiencies *vs.* Luminance relationships.


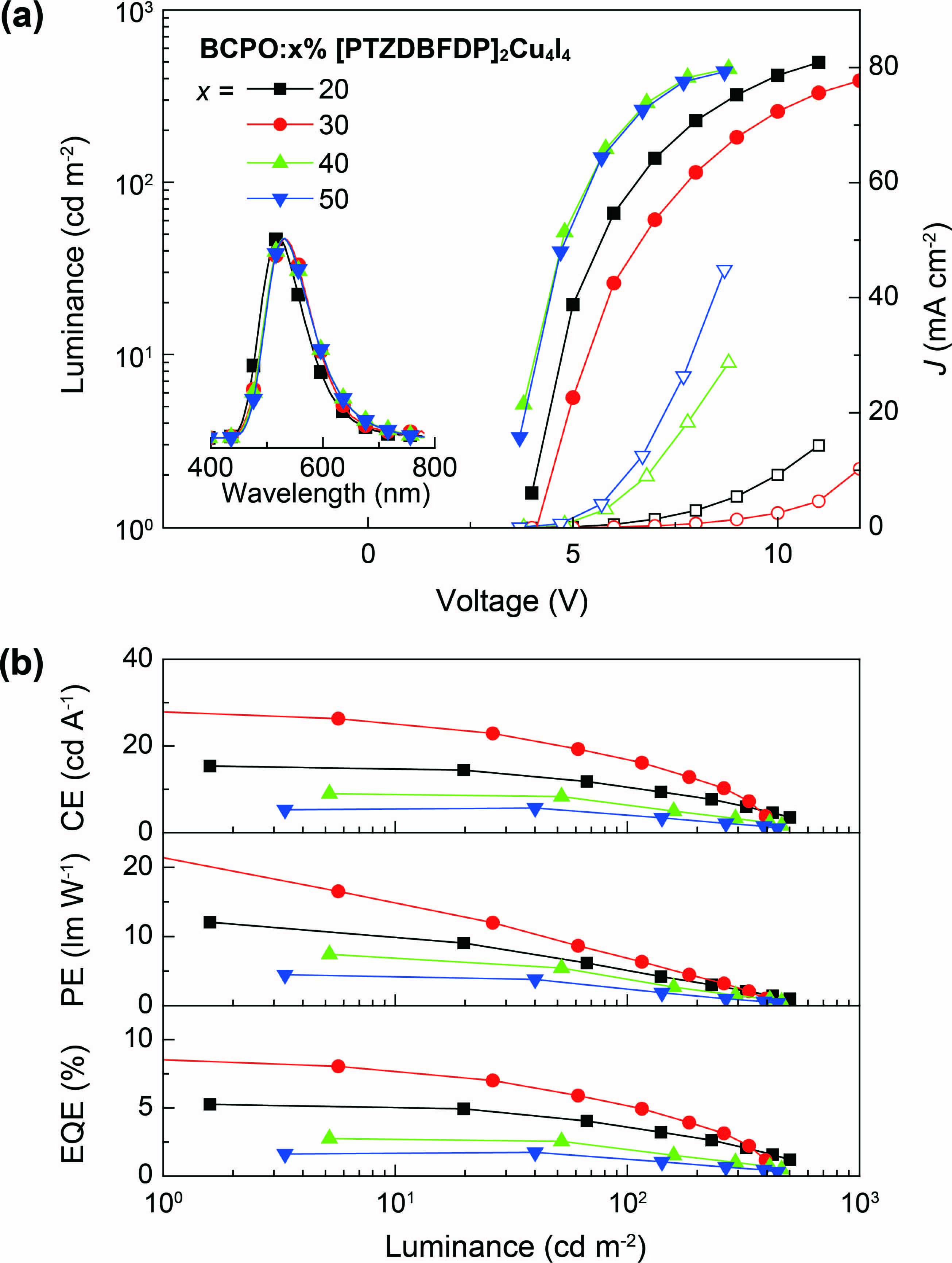


**Figure S12.** (**a**) EL spectra (inset) and Current density (*J*)-Voltage-Luminance characteristics of BCPO:*x*% **[PTZDBFDP]_2_Cu_4_I_4_** based CLEDs at different *x*; (**b**) Efficiencies *vs.* Luminance relationships.


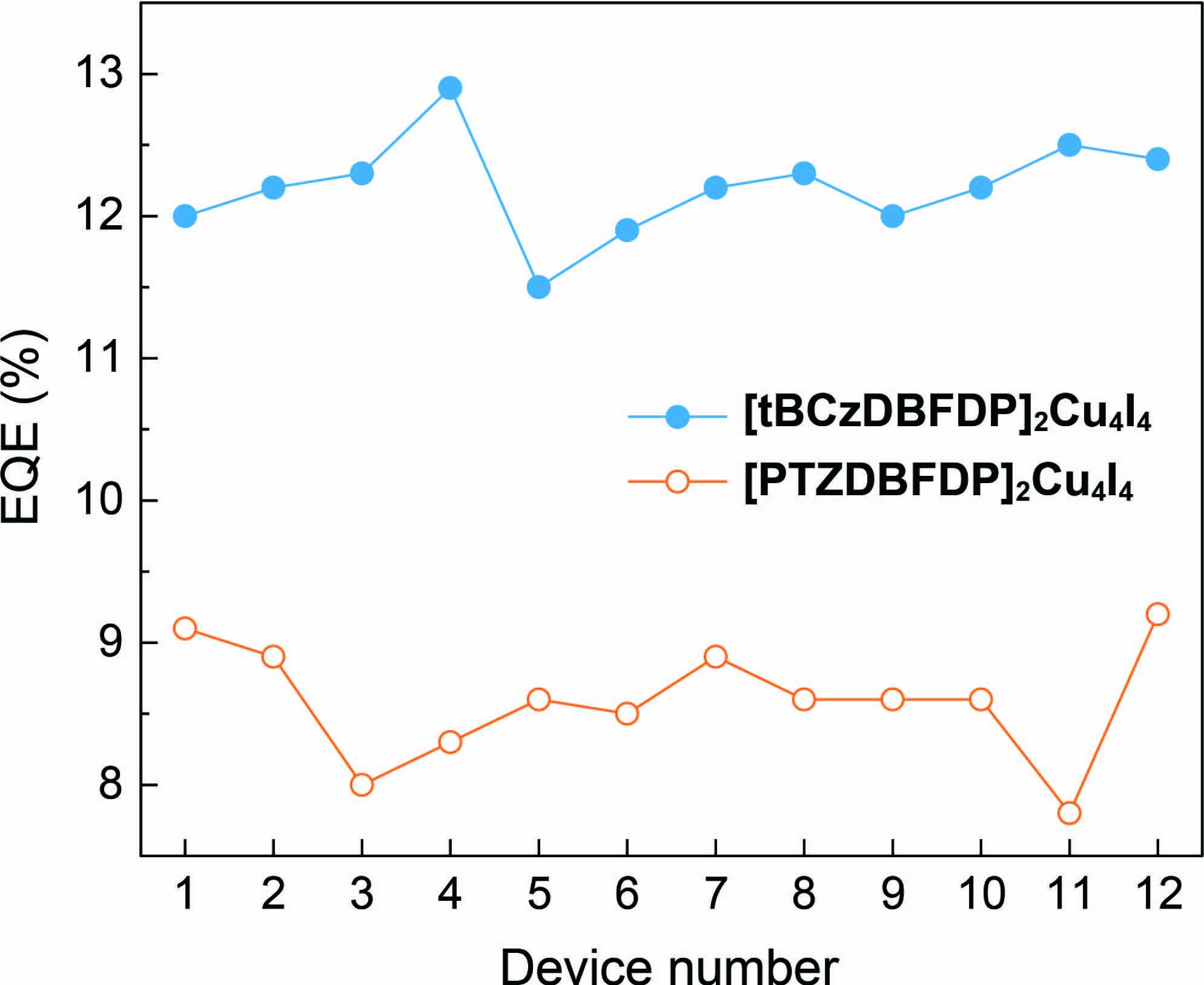


**Figure S13.** EQE distributions of 12 parallel devices based on **[tBCzDBFDP]_2_Cu_4_I_4_** and **[PTZDBFDP]_2_Cu_4_I_4_**.

### Table S2. EL performance of CLEDs based on the clusters.

| EML | | *x* (wt%) | *V*^[a]^ (V) | *L*_max_^[b]^  (cd m^-2^) | *η*^[c]^ | | | *λ*_EL_ (nm) /  CIE (x, y)^[d]^ |
| --- | --- | --- | --- | --- | --- | --- | --- | --- |
|  |  |  |  |  | *η*_CE_ (cd A^-1^) | *η*_PE_ (lm W^-1^) | *η*_EQE_ (%) |  |
| **BCPO:*x*% [tBCzDBFDP]_2_Cu_4_I_4_** | 20 | | 4.0, 5.9, 9.7 | 1247 | 21.5, 18.4 6.7 | 16.9, 9.7, 2.1 | 12.2, 7.3, 2.7 | 492/(0.20, 0.41) |
|  | 30 | | 3.7, 5.2, 7.8 | 1709 | 28.1, 12.6, 4.2 | 23.9, 20.6, 10.5 | 11.0, 8.1, 2.7 | 496/(0.21, 0.43) |
|  | 40 | | 3.5, 4.9, 6.9 | 1477 | 29.3, 22.4, 12.5 | 26.3, 14.8, 5.9 | 11.4, 8.8, 4.9 | 500/(0.22, 0.44) |
|  | 50 | | 3.7, 4.7, 7.1 | 1193 | 20.3, 17.0, 8.0 | 17.2, 11.4, 3.6 | 7.9, 6.6, 3.1 | 504/(0.23, 0.45) |
| **BCPO:*x*% [PTZDBFDP]_2_Cu_4_I_4_** | 20 | | 4.0, 6.5, - | 501 | 15.4, 10.5, - | 12.1, 5.0, - | 5.2, 3.4, - | 520/(0.30, 0.55) |
|  | 30 | | 4.0, 7.6, - | 394 | 28.1, 16.6, - | 22.0, 7.0, - | 8.6, 4.9, - | 532/(0.34, 0.56) |
|  | 40 | | 3.8, 5.4, - | 462 | 9.0, 6.7, - | 7.4, 3.7, - | 2.7, 2.0, - | 532/(0.34, 0.56) |
|  | 50 | | 3.7, 5.5, - | 444 | 5.7, 3.8, - | 4.8, 2.3, - | 1.7, 1.3, - | 532/(0.34, 0.56) |

[a] At 1, 100 and 1000 cd m^-2^; [b] the maximum luminance; [c] EL efficiencies at the maximum, 100 and 1000 cd m^-2^; [d] peak wavelengths and CIE coordinates of EL emissions at 1000 cd m^-2^.

**Table S3.** Comparison on performances of representative EL clusters.

| Cluster | *ϕ*_PL_^[a]^ (%) | *V*^[a]^ (V) | *L*_max_^[b]^  (cd m^-2^) | *η*^[c]^ | | | *λ*_EL_^[d]^  (nm) | CIE^[e]^ |
| --- | --- | --- | --- | --- | --- | --- | --- | --- |
|  |  |  |  | *η*_CE_ (cd A^-1^) | *η*_PE_ (lm W^-1^) | *η*_EQE_ (%) |  |  |
| **(TBA)_2_Mo_6_Cl_14_**^(^*^5^*^)^ | >70 | - | - | -, -, - | -, -, - | 0.003, -, - | 790 | -, - |
| **[DBFDP]_2_Cu_4_I_4_**_(_*_6_*_)_ | ~5 | 7.5, 10.0, 14.0 | ~1500 | 1.8, 1.8, 0.9 | 0.5, 0.5, 0.2 | 0.7, 0.7, 0.4 | 550 | 0.37, 0.45 |
| **[2][BAr^F^_4_]**^(^*^7^*^)^ | 93 | 2.4, -, - | ~19000 | -, -, - | -, -, - | 11.2, -, - | 525 | 0.31, 0.64 |
| **[DtBCzDBFDP]_2_Cu_4_I_4_**^(^*^8^*^)^ | 65 | 6.0, 10.0, 13.0 | 6772 | 20.2, 15.7, 12.7 | 10.6, 6.1, 3.1 | 7.9, 6.1, 5.0 | 492 | 0.23, 0.42 |
| **Cu_2_I_2_(BINAP)_2_**^(^*^9^*^)^ | 4.7 | 2.9, -, - | ~1200 | -, -, - | -, -, - | 0.54, -, - | 520 | -, - |
| **[DMACDBFDP]_2_Cu_4_I_4_**^(^*^10^*^)^ | 73 | 3.3, 4.4, 5.9 | 4016 | 41.9, 38.8, 26.8 | 39.9, 27.8, 14.6 | 15.7, 14.5, 10.0 | 500 | 0.22, 0.45 |
| **[DPACDBFDP]_2_Cu_4_I_4_**^(^*^10^*^)^ | 81 | 3.5, 4.7, 6.2 | 4440 | 49.4, 43.4, 28.6 | 44.3, 29.3, 14.6 | 19.5, 16.6, 11.0 | 500 | 0.21, 0.44 |
| **[tBCzDBFDP]_2_Cu_4_I_4_** | 68 | 4.0, 5.9, 9.7 | 1247 | 21.5, 18.4 6.7 | 16.9, 9.7, 2.1 | 12.2, 7.3, 2.7 | 492 | 0.20, 0.41 |
| **[PTZDBFDP]_2_Cu_4_I_4_** | 45 | 4.0, 7.6, - | 394 | 28.1, 16.6, - | 22.0, 7.0, - | 8.6, 4.9, - | 532 | 0.34, 0.56 |

[a] Photoluminescent quantum yield in film; [b] At 1, 100 and 1000 cd m^-2^; [c] the maximum luminance; [d] EL efficiencies at the maximum, 100 and 1000 cd m^-2^; [e] EL emission peak wavelength; [f] Commission Internationale de lEclairage 1931 chromatic system.

**
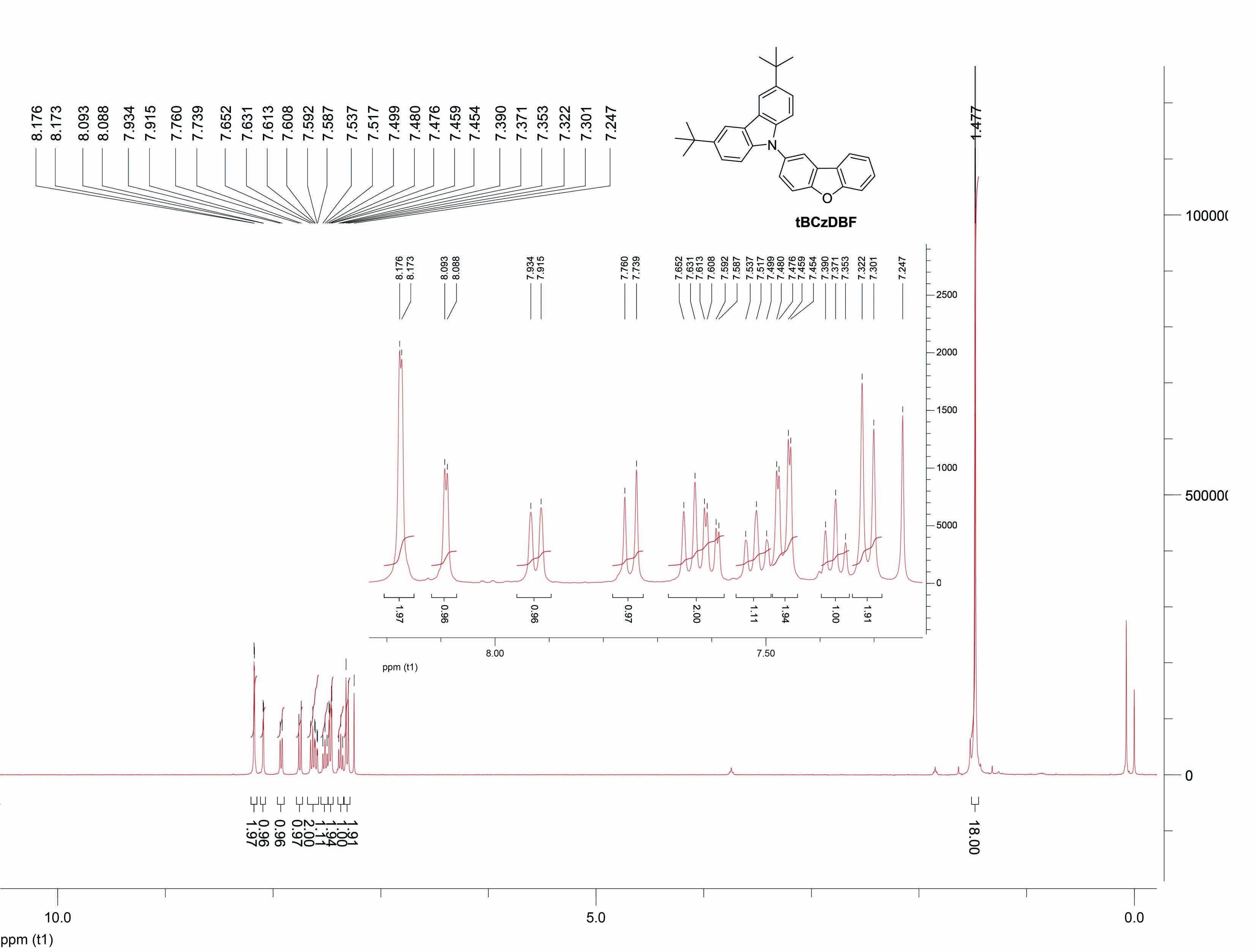
**

**Figure S14.** ^1^H NMR spectrum of tBCzDBF in CDCl_3_.

**
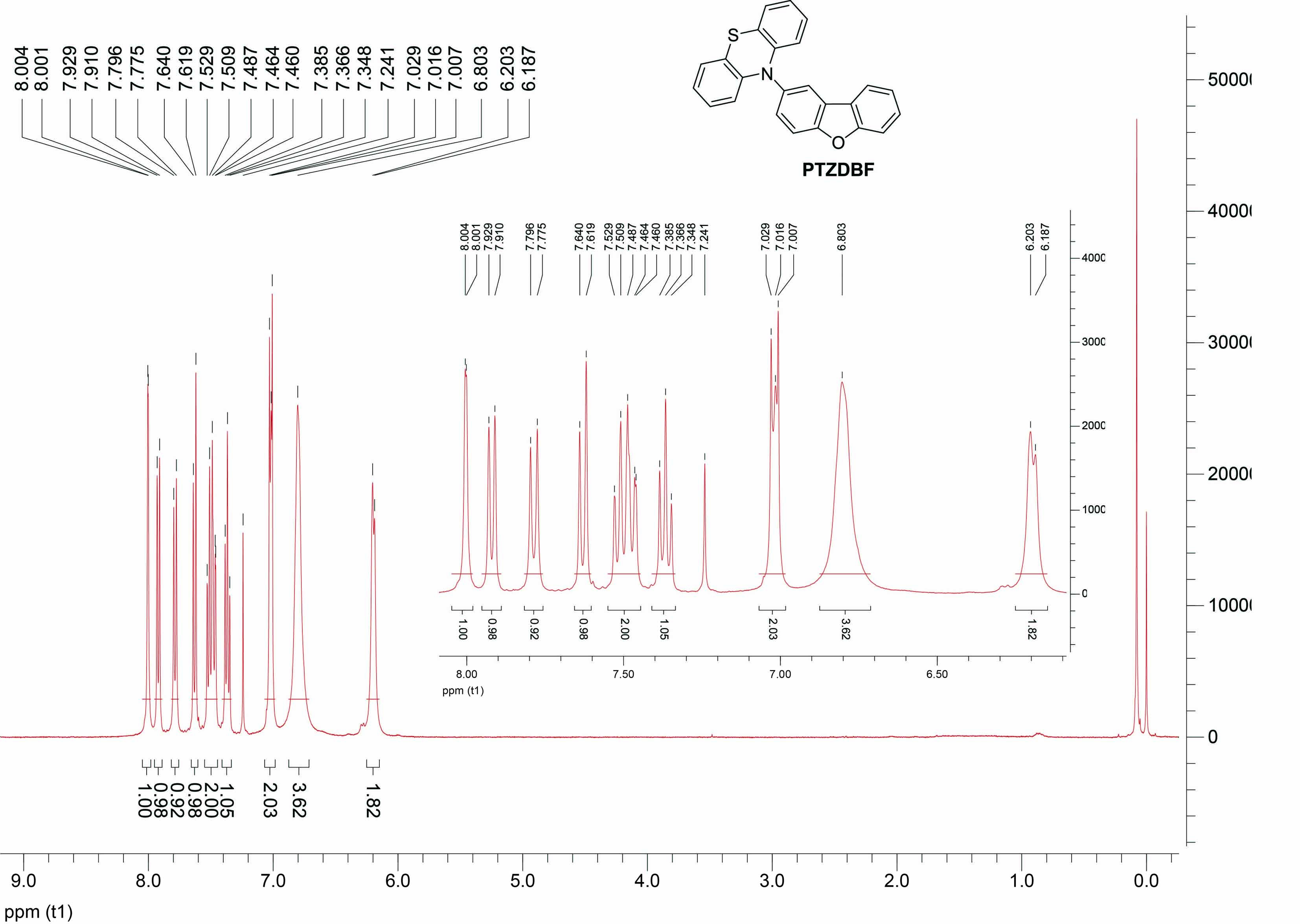
**

**Figure S15.** ^1^H NMR spectrum of PTZDBF in CDCl_3_.

**
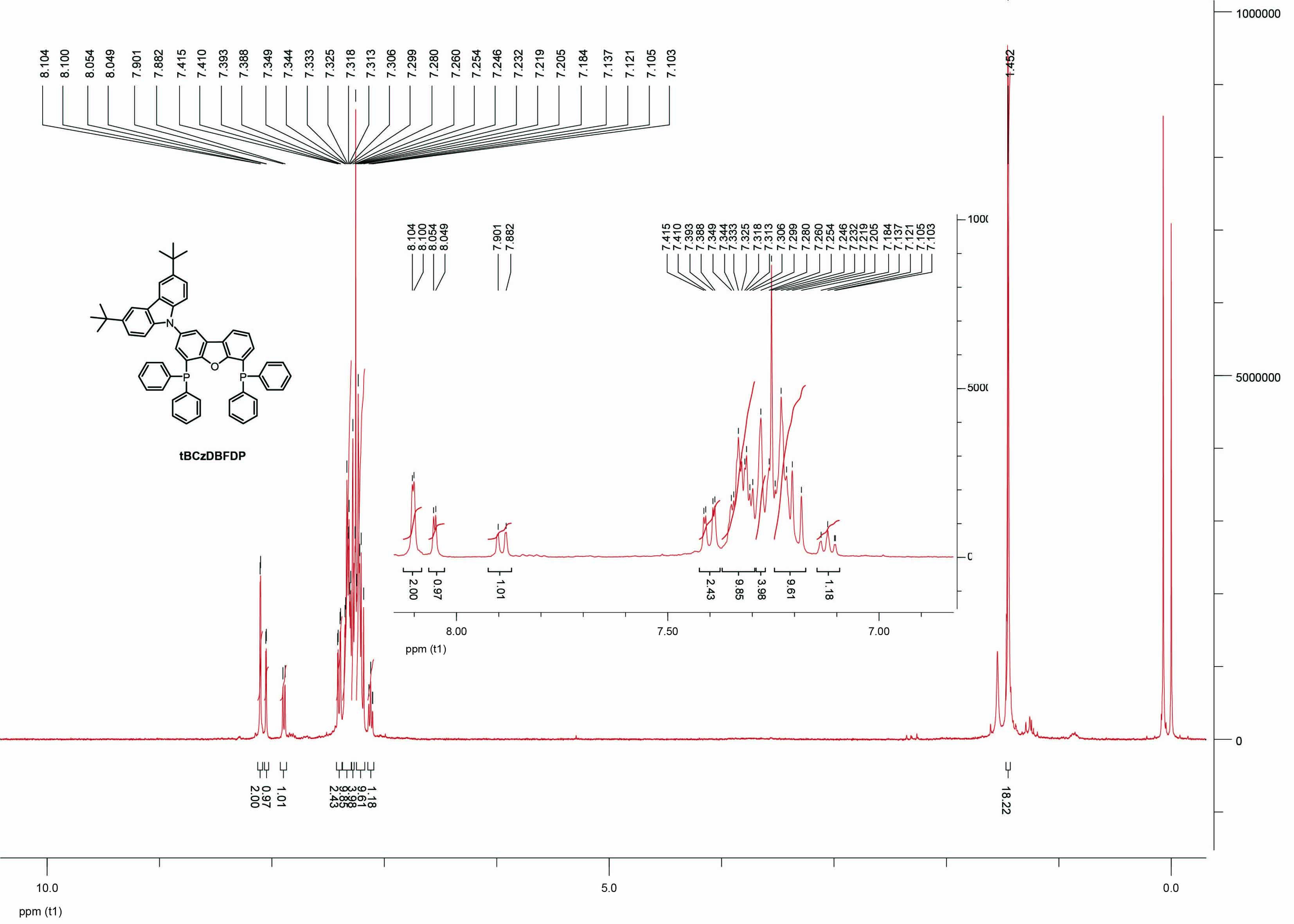
**

**Figure S16.** ^1^H NMR spectrum of tBCzDBFDF in CDCl_3_.

**
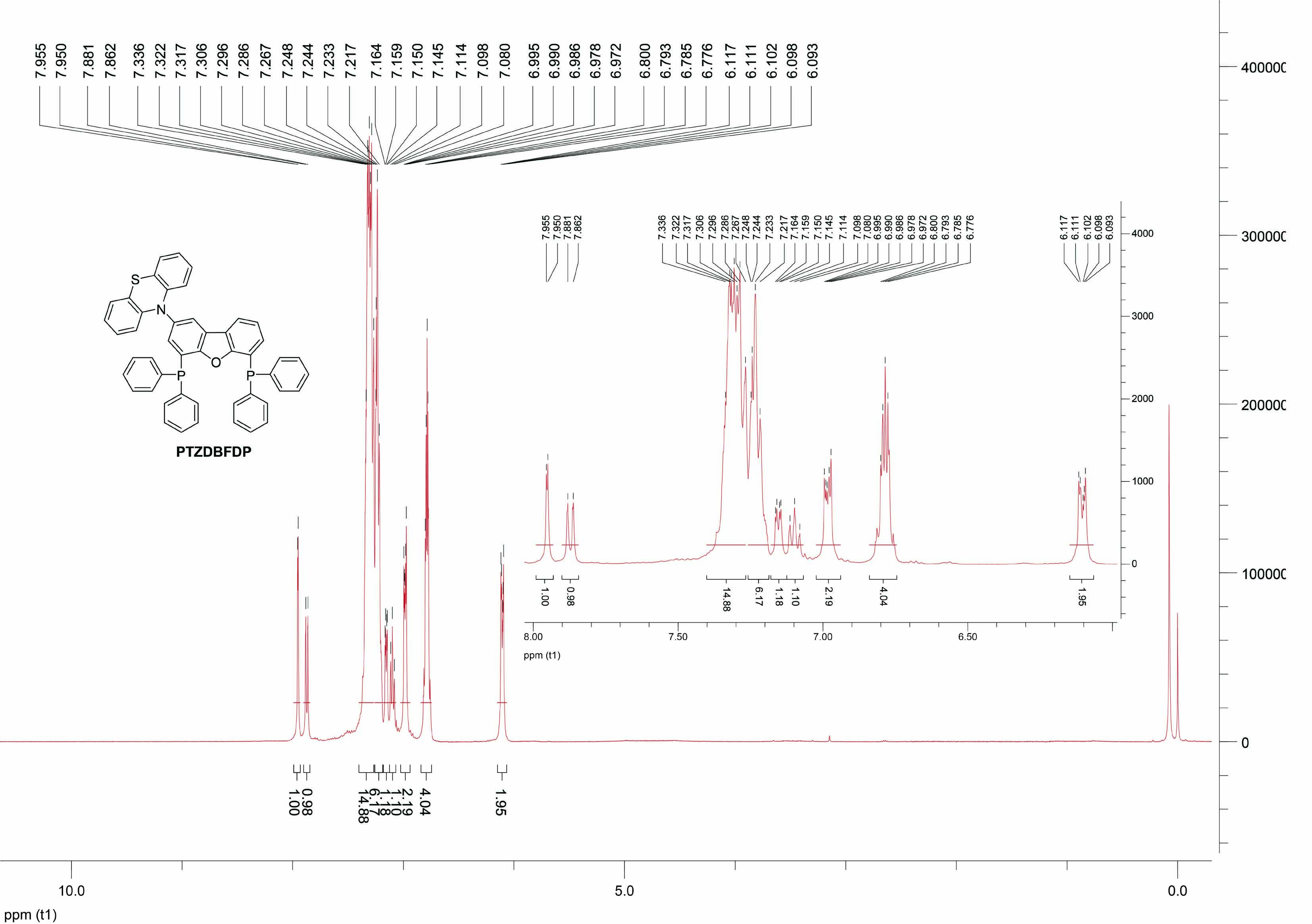
**

**Figure S17.** ^1^H NMR spectrum of PTZDBFDF in CDCl_3_.

**
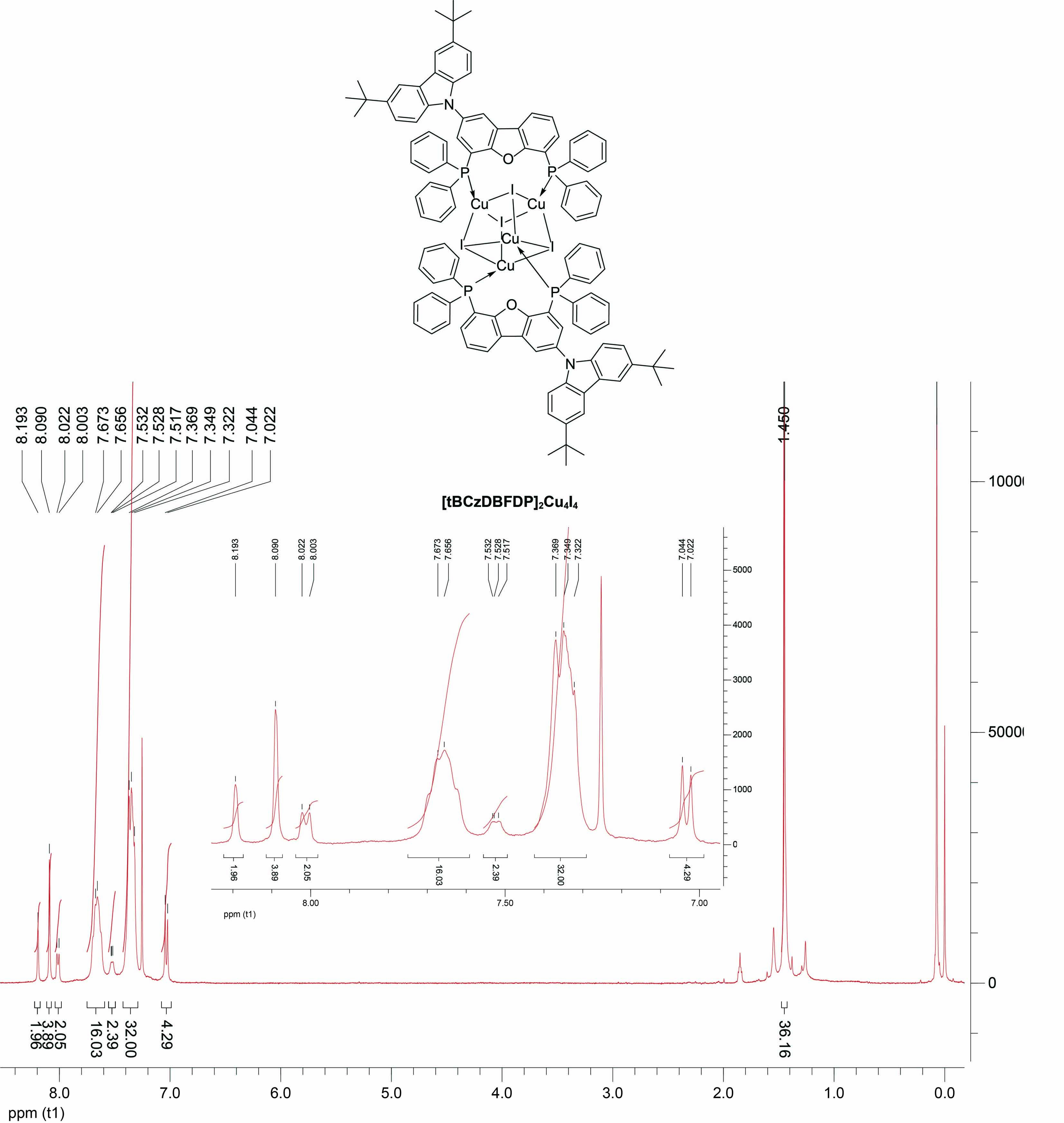
**

**Figure S18.** ^1^H NMR spectrum of [tBCzDBFDF]_2_Cu_4_I_4_ in CDCl_3_.

**
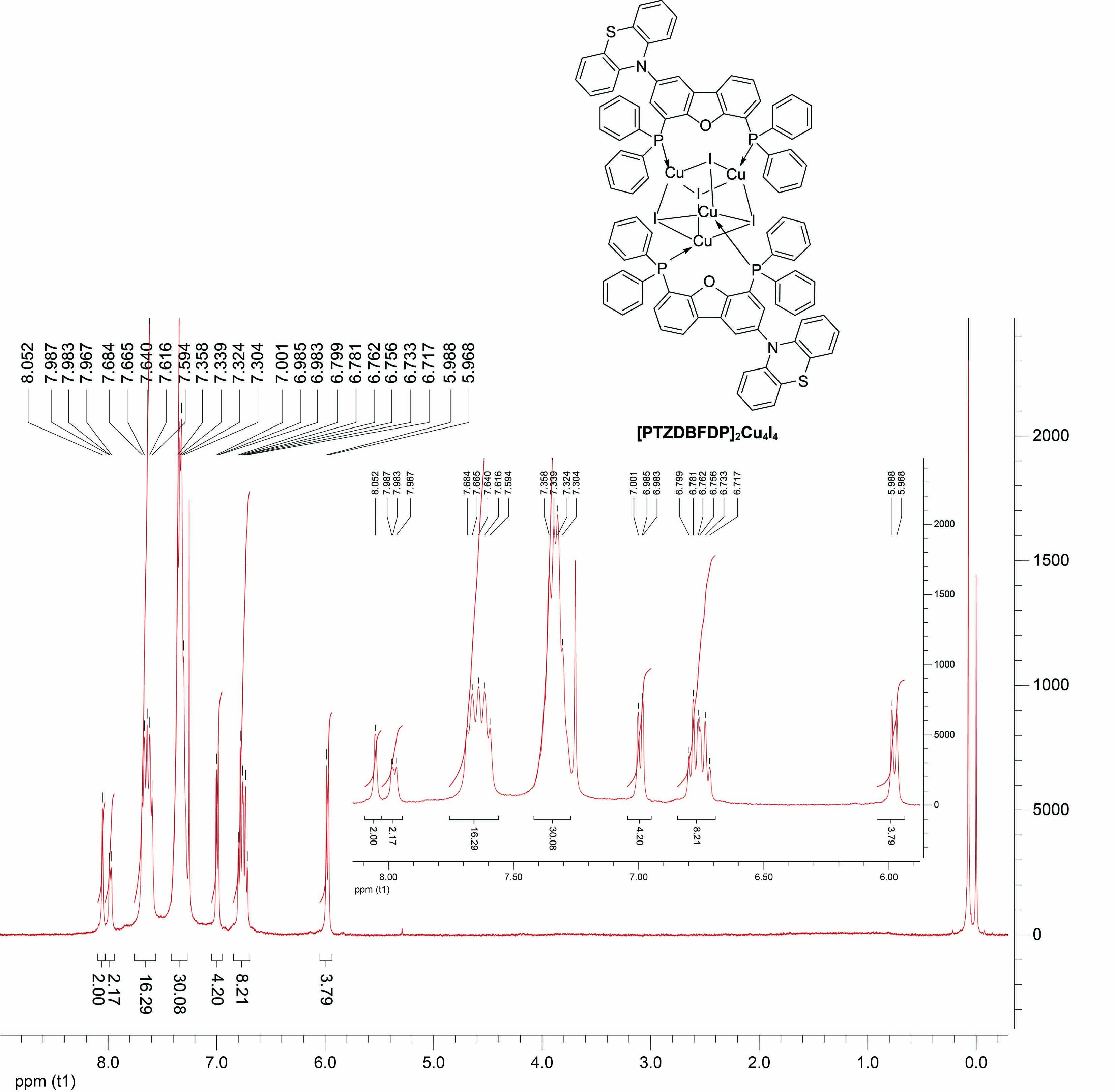
**

**Figure S19.** ^1^H NMR spectrum of [PTZDBFDF]_2_Cu_4_I_4_ in CDCl_3_.

**
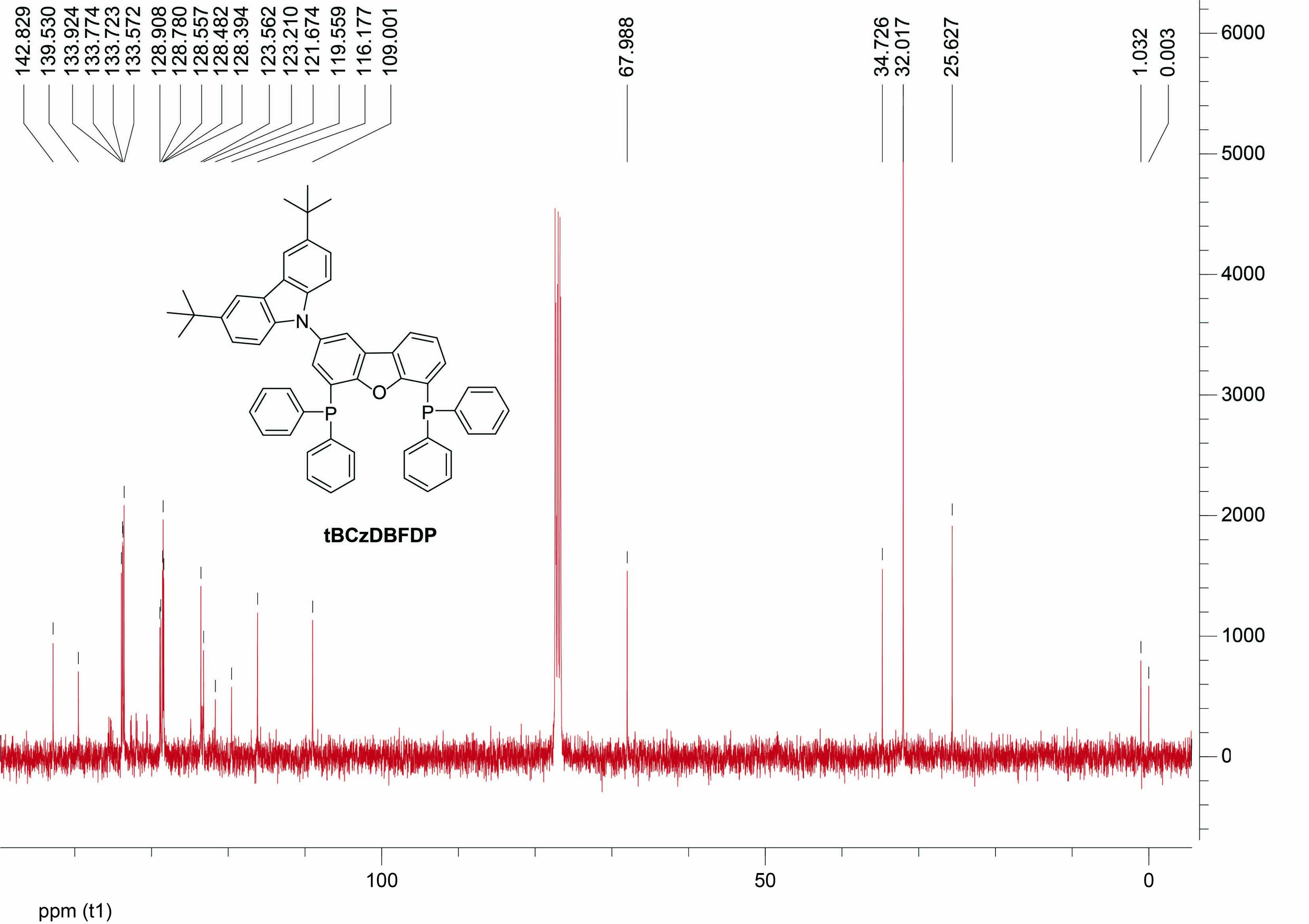
**

**Figure S20.** ^13^C NMR spectrum of tBCzDBFDP in CDCl_3_.

**
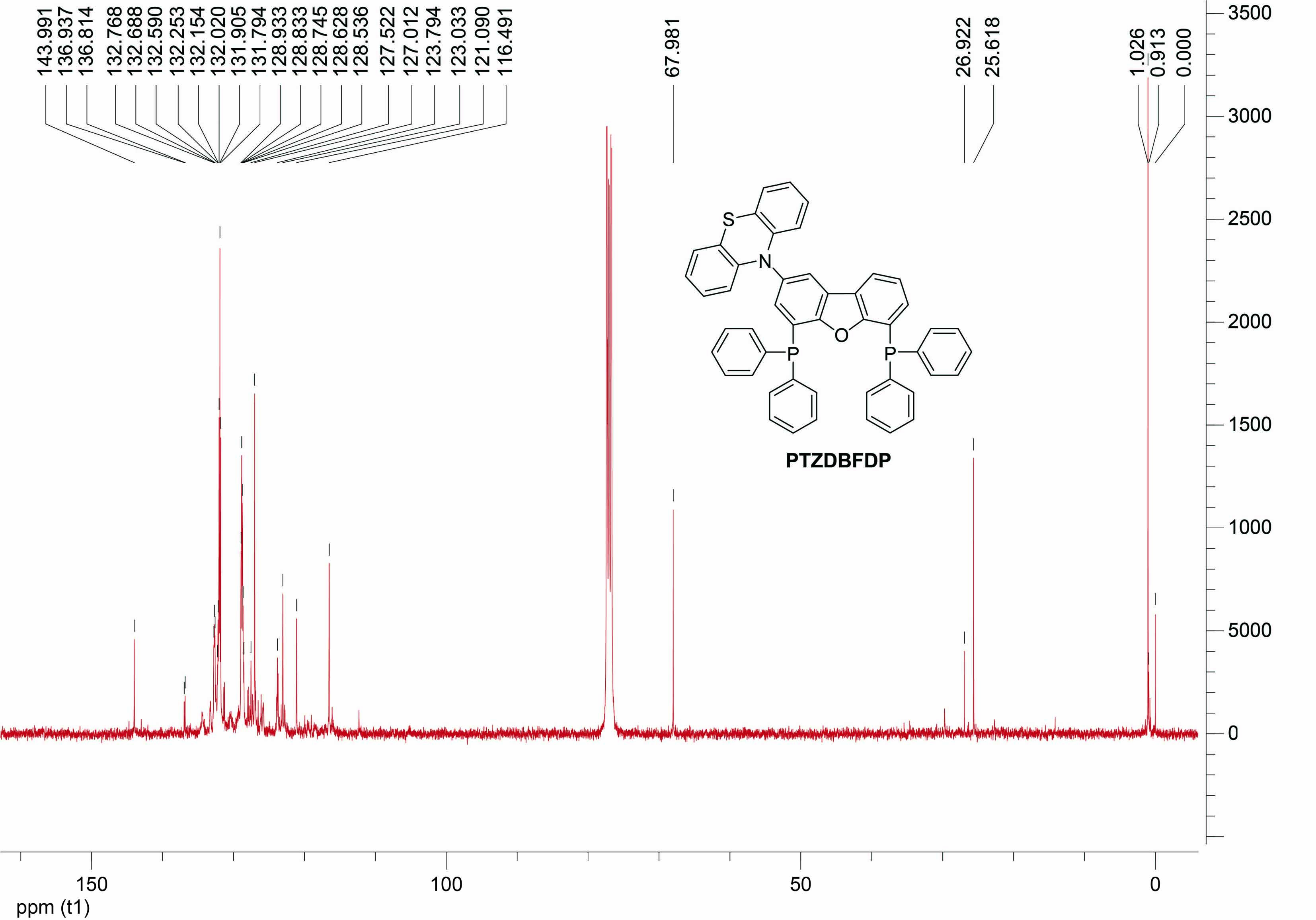
**

**Figure S21.** ^13^C NMR spectrum of PTZDBFDP in CDCl_3_.

**
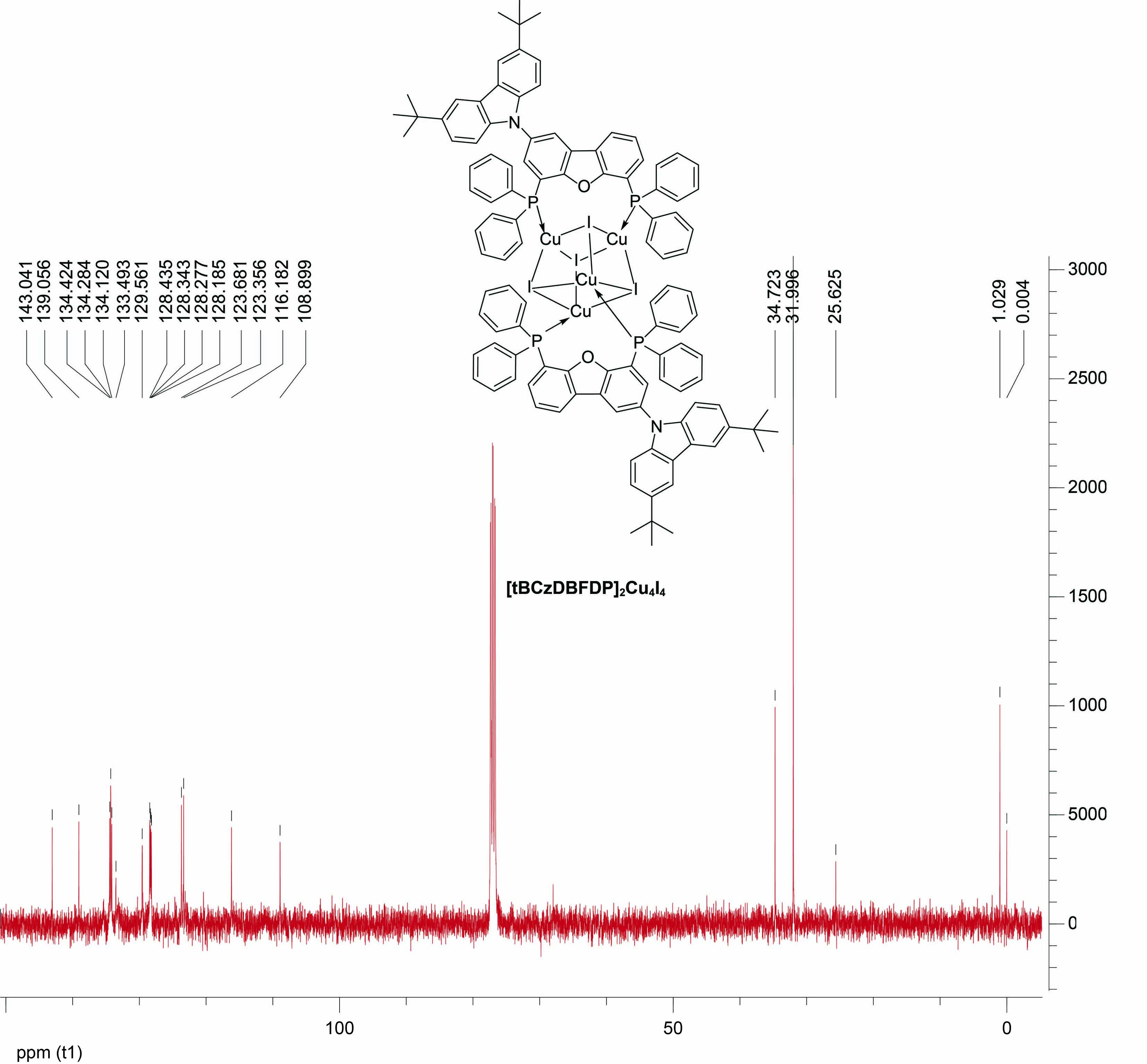
**

**Figure S22.** ^13^C NMR spectrum of [tBCzDBFDF]_2_Cu_4_I_4_ in CDCl_3_.

**
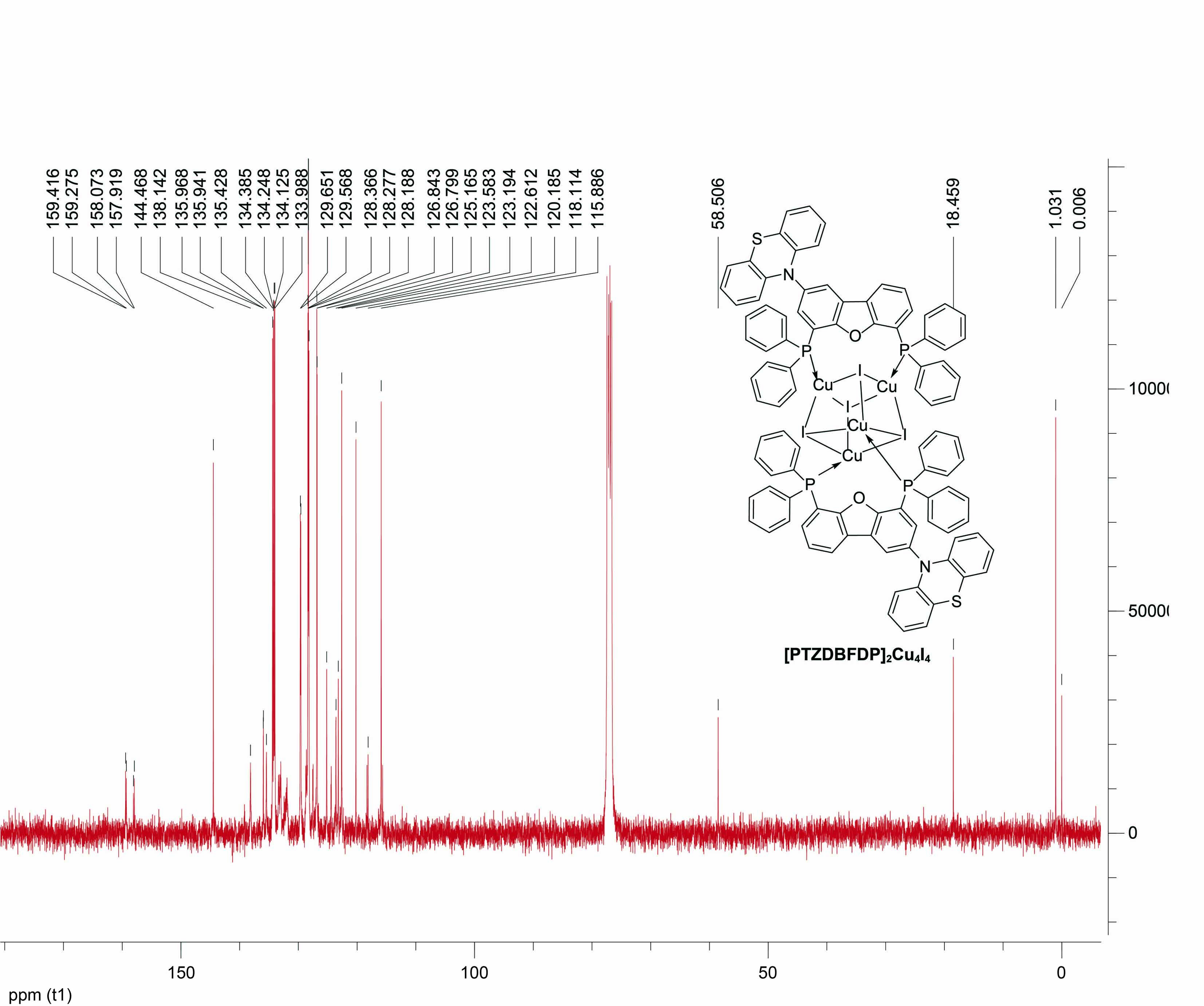
**

**Figure S23.** ^13^C NMR spectrum of [PTZDBFDF]_2_Cu_4_I_4_ in CDCl_3_.

**
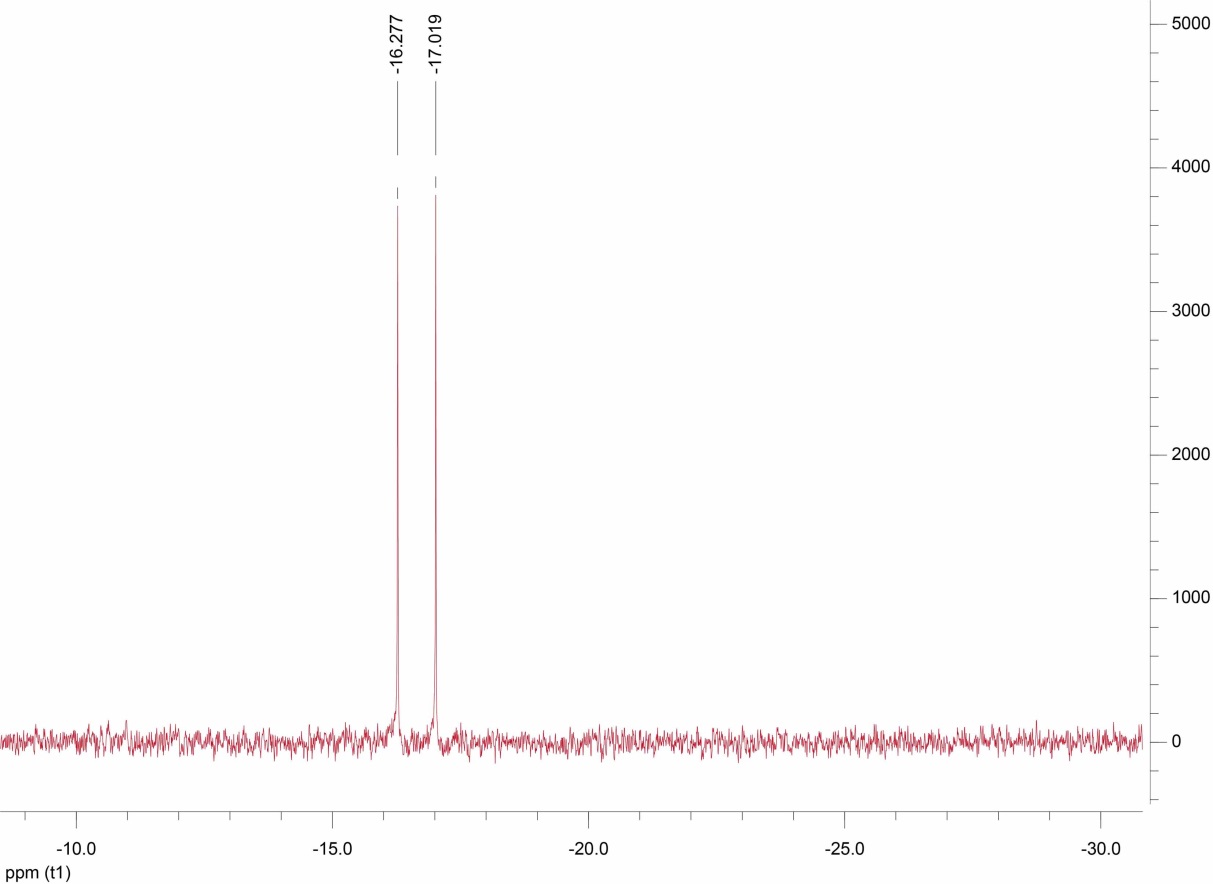
**

**Figure S24.** ^31^P NMR spectrum of tBCzDBFDP in CDCl_3_.

**
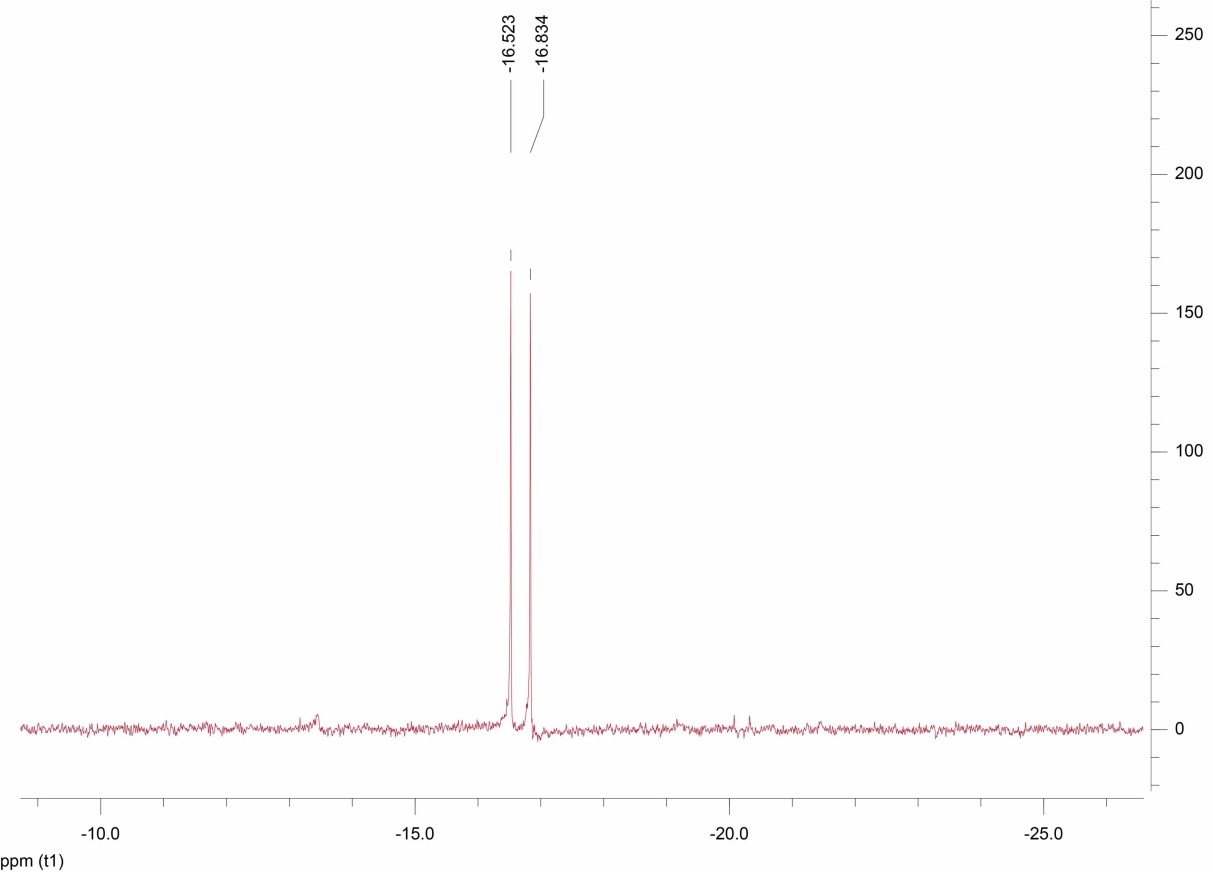
**

**Figure S25.** ^31^P NMR spectrum of PTZDBFDP in CDCl_3_.

**
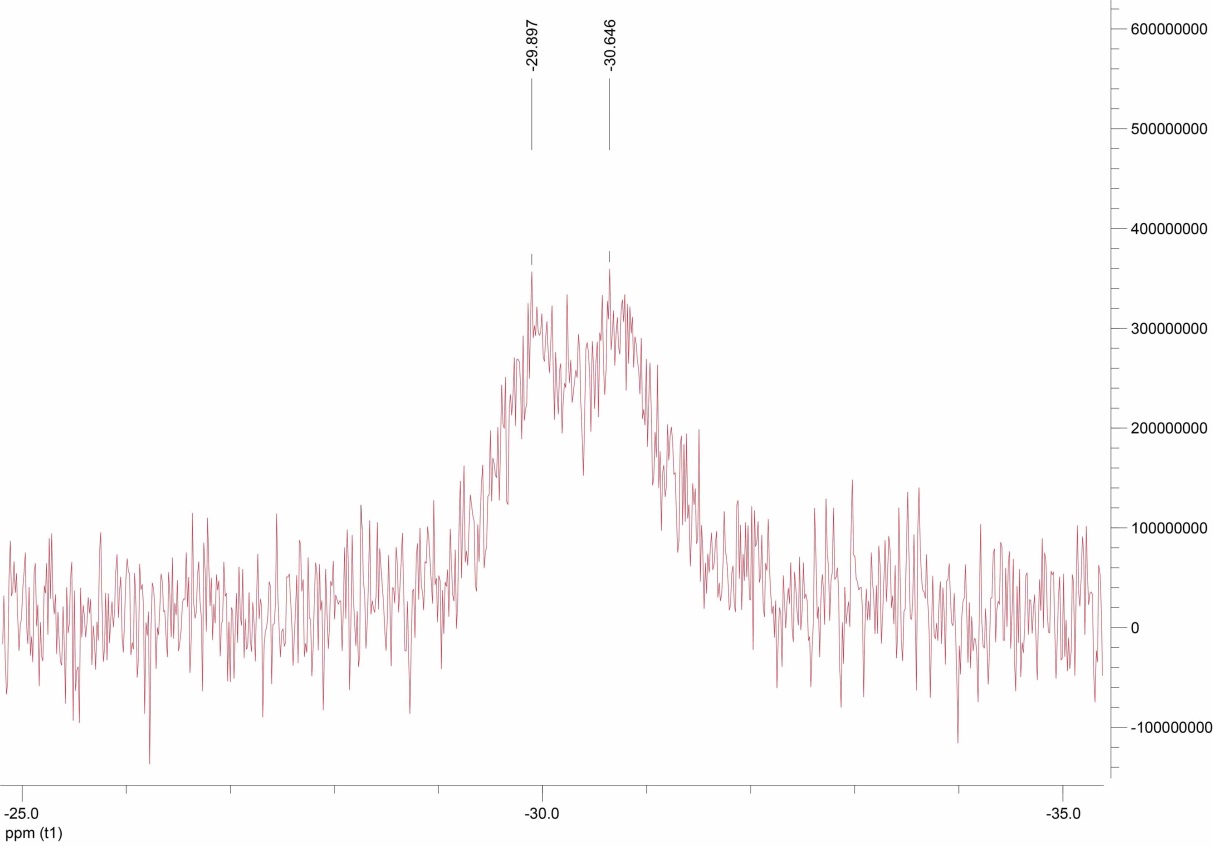
**

**Figure S26.** ^31^P NMR spectrum of [tBCzDBFDF]_2_Cu_4_I_4_ in CDCl_3_.

**
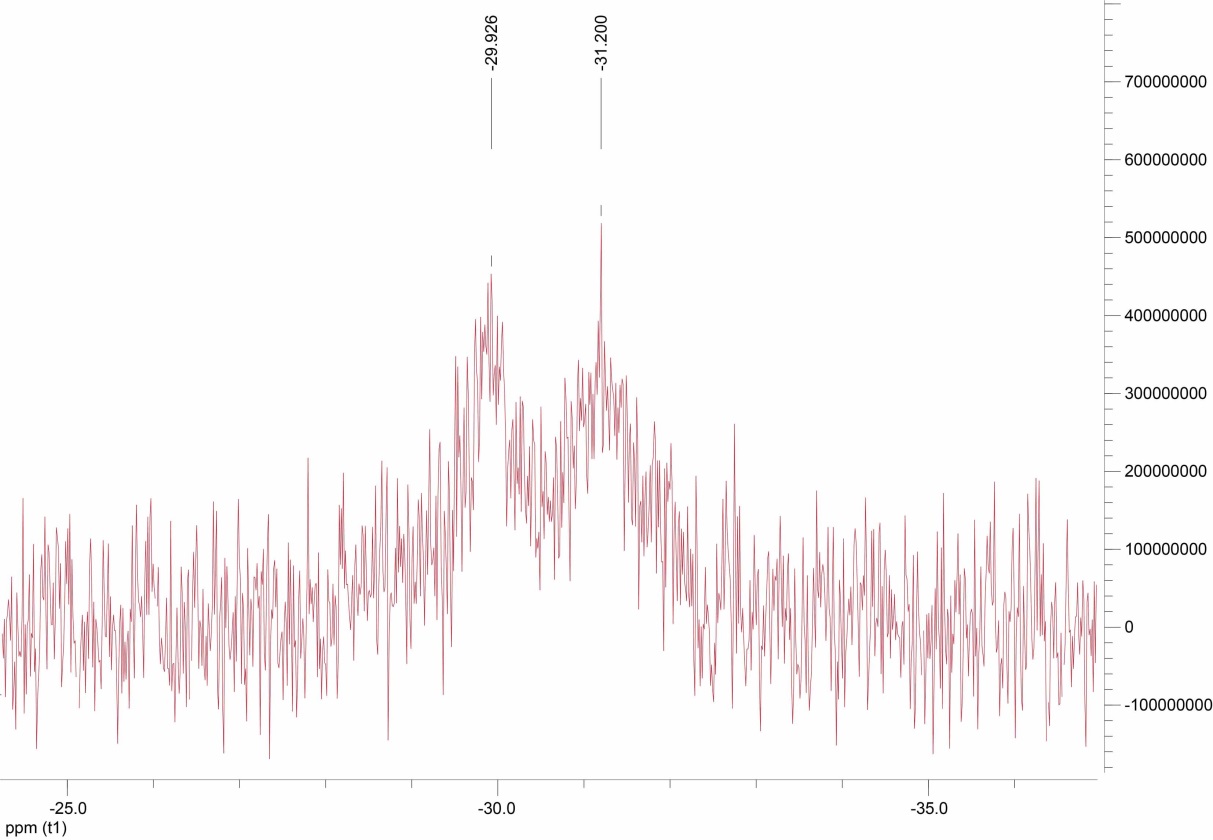
**

**Figure S27.** ^31^P NMR spectrum of [PTZDBFDF]_2_Cu_4_I_4_ in CDCl_3_.

### References

1. A. D. Becke, Density-functional thermochemistry. III. The role of exact exchange. *J. Chem. Phys.* **98**, 5648-5652 (1993).

2. C. Lee, W. Yang, R. G. Parr, Development of the Colle-Salvetti correlation-energy formula into a functional of the electron density. *Phys. Rev. B* **37**, 785-789 (1988).

3. R. L. Martin, Natural transition orbitals. *J. Chem. Phys.* **118**, 4775-4777 (2003)10.1063/1.1558471).

4. M. J. Frisch, G. W. Trucks, H. B. Schlegel, G. E. Scuseria, M. A. Robb, J. R. Cheeseman, G. Scalmani, V. Barone, B. Mennucci, G. A. Petersson, H. Nakatsuji, M. Caricato, X. Li, H. P. Hratchian, A. F. Izmaylov, J. Bloino, G. Zheng, J. L. Sonnenberg, M. Hada, M. Ehara, K. Toyota, R. Fukuda, J. Hasegawa, M. Ishida, T. Nakajima, Y. Honda, O. Kitao, H. Nakai, T. Vreven, J. A. Montgomery, J. E. Peralta, F. Ogliaro, M. Bearpark, J. J. Heyd, E. Brothers, K. N. Kudin, V. N. Staroverov, R. Kobayashi, J. Normand, K. Raghavachari, A. Rendell, J. C. Burant, S. S. Iyengar, J. Tomasi, M. Cossi, N. Rega, J. M. Millam, M. Klene, J. E. Knox, J. B. Cross, V. Bakken, C. Adamo, J. Jaramillo, R. Gomperts, R. E. Stratmann, O. Yazyev, A. J. Austin, R. Cammi, C. Pomelli, J. W. Ochterski, R. L. Martin, K. Morokuma, V. G. Zakrzewski, G. A. Voth, P. Salvador, J. J. Dannenberg, S. Dapprich, A. D. Daniels, Ö. Farkas, J. B. Foresman, J. V. Ortiz, J. Cioslowski, D. J. Fox. (Gaussian, Inc., Wallingford CT, USA, 2009).

5. P. S. Kuttipillai, Y. Zhao, C. J. Traverse, R. J. Staples, B. G. Levine, R. R. Lunt, Phosphorescent Nanocluster Light-Emitting Diodes. *Adv. Mater.* **28**, 320-326 (2016)10.1002/adma.201504548).

6. M. Xie, C. Han, J. Zhang, G. Xie, H. Xu, White Electroluminescent Phosphine-Chelated Copper Iodide Nanoclusters. *Chem. Mater.* **29**, 6606-6610 (2017); published online Epub2017/08/22 (10.1021/acs.chemmater.7b01443).

7. M. Olaru, E. Rychagova, S. Ketkov, Y. Shynkarenko, S. Yakunin, M. V. Kovalenko, A. Yablonskiy, B. Andreev, F. Kleemiss, J. Beckmann, M. Vogt, A Small Cationic Organo–Copper Cluster as Thermally Robust Highly Photo- and Electroluminescent Material. *J. Am. Chem. Soc.* **142**, 373-381 (2020); published online Epub2020/01/08 (10.1021/jacs.9b10829).

8. M. Xie, C. Han, Q. Liang, J. Zhang, G. Xie, H. Xu, Highly efficient sky blue electroluminescence from ligand-activated copper iodide clusters: Overcoming the limitations of cluster light-emitting diodes. *Sci. Adv.* **5**, eaav9857 (2019)10.1126/sciadv.aav9857).

9. J.-J. Wang, H.-T. Zhou, J.-N. Yang, L.-Z. Feng, J.-S. Yao, K.-H. Song, M.-M. Zhou, S. Jin, G. Zhang, H.-B. Yao, Chiral Phosphine–Copper Iodide Hybrid Cluster Assemblies for Circularly Polarized Luminescence. *J. Am. Chem. Soc.* **143**, 10860-10864 (2021); published online Epub2021/07/28 (10.1021/jacs.1c05476).

10. N. Zhang, H. Hu, L. Qu, R. Huo, J. Zhang, C. Duan, Y. Meng, C. Han, H. Xu, Overcoming Efficiency Limitation of Cluster Light-Emitting Diodes with Asymmetrically Functionalized Biphosphine Cu4I4 Cubes. *J. Am. Chem. Soc.* **144**, 6551-6557 (2022); published online Epub2022/04/13 (10.1021/jacs.2c01588).
